# CD44 promotes angiogenesis in myocardial infarction through regulating plasma exosome uptake and further enhancing FGFR2 signaling transduction

**DOI:** 10.1186/s10020-022-00575-5

**Published:** 2022-12-03

**Authors:** Qing Zhang, Li Chen, Liyi Huang, Hongxin Cheng, Lu Wang, Lin Xu, Danrong Hu, Chengqi He, Chenying Fu, Quan Wei

**Affiliations:** 1 Rehabilitation Medicine Center and Institute of Rehabilitation Medicine, West China Hospital, Sichuan University, Chengdu, Sichuan People’s Republic of China; 2 Key Laboratory of Rehabilitation Medicine in Sichuan Province, Chengdu, Sichuan People’s Republic of China; 3 Department of Rehabilitation Medicine, The Fifth Affiliated People’s Hospital of Chengdu University of Traditional Chinese Medicine, Chengdu, Sichuan People’s Republic of China; 4 National Clinical Research Center for Geriatrics, West China Hospital, Sichuan University, Chengdu, Sichuan People’s Republic of China; 5 Aging and Geriatric Mechanism Laboratory, West China Hospital, Sichuan University, Chengdu, Sichuan People’s Republic of China

**Keywords:** CD44, Angiogenesis, Exosome uptake, FGFR2 signaling pathway, Myocardial infarction

## Abstract

**Background:**

Since angiogenesis occurs as the pathological process following myocardial infarction to alleviate ischemia, therapeutic angiogenesis has been proposed to be a cardioprotective strategy. CD44 has been implicated in endothelial cell functions and its role has been well established in angiogenesis for years. Although recent studies indicate the close correlation between CD44 and exosome, as well as the two being implicated in myocardial ischemia pathological processes, the effect and the underlying mechanism of CD44 and its regulated plasma exosome in pathological angiogenesis post-myocardial infarction have not been fully elucidated.

**Methods:**

In this study, we used CD44 knockout mice to study the in vivo impacts of CD44 on ischemic angiogenesis in myocardial infarction. Mouse cardiac function was measured by echocardiography, histological changes were observed by Evans Blue and TTC-double staining and Masson’s trichrome staining, and molecular changes were detected by immunofluorescence. In the in vitro study, CD44 knockout HUVECs were generated and CD44 inhibitor was used to study the mechanism of CD44 on angiogenesis. We performed the immunoprecipitation, proximity ligation assay, and super-resolution imaging to study the mechanistic regulation of FGFR2 signaling transduction by CD44. Importantly, we also isolated plasma exosomes from myocardial infarction model mice and studied the effect of plasma exosomes on the activation of the FGFR2 signaling pathway and the related phenotypic alterations, including exosomes uptake and angiogenic function in primary mouse microvascular endothelial cells, and further discovered the regulation mechanism of exosomal miRNAs.

**Results:**

We observed that the expression of CD44 in the border zone of the infarcted heart was tightly related to pathological angiogenesis following myocardial ischemia. The depletion of CD44 impaired angiogenesis and impacts biogenesis and proangiogenic function of plasma exosomes. Subsequently, we found that CD44 mediated the activation of the FGFR2 signaling pathway as well as the caveolin 1-dependent uptake of exosomes in vascular endothelial cells. Most importantly, the proangiogenic therapeutic effect of plasma exosomal miRNAs depended upon the participation of CD44/FGFR2 signaling transduction in vascular endothelial cells.

**Conclusion:**

CD44 and its regulated plasma exosomes have crucial potent angiogenic activity. Our studies elucidate that CD44 plays a key role in plasma exosomal miRNA-enhanced angiogenic FGFR2 singling transduction and ischemic angiogenesis in the early stage of myocardial infarction.

**Supplementary Information:**

The online version contains supplementary material available at 10.1186/s10020-022-00575-5.

## Introduction

The increasing morbidity and mortality of cardiovascular disease, especially ischemic heart disease causes a heavy burden on medical care worldwide (Roth et al 2020; Roth et al. 2020). The most common cause of acute myocardial infarction (AMI) is the rupture of coronary atherosclerotic plaque in patients with coronary heart disease, forming arterial embolism and blocking blood supply (Khera and Kathiresan, 2017). Herein, therapies that aim to enhance angiogenesis may be beneficial to relieve heart tissue ischemia and reduce the mortality of patients. Therapeutic angiogenesis was proposed in this regard, which refers to a method to promote angiogenesis and restore blood perfusion in ischemic myocardium (Losordo and Dimmeler 2004a, 2004b; Mitsos et al. 2012). Growing evidence has recently highlighted the perspective of novel targeted strategies for therapeutic angiogenesis in MI, including exosome therapy (Sahoo and Losordo 2014;  Zhang et al. 2022). Exosomes, lipid bilayer vesicles, carry and transfer diverse biomolecules that have been shown to be promising tools in the treatment of myocardial ischemia (Sahoo et al. 2021; Sahoo and Losordo 2014). In particular, endogenous exosomes excreted into blood, which convey biosignals throughout the body, accumulate in the injured myocardium and act as cardiac protectors (Lassen et al. 2021; Liu et al. 2020; Vicencio et al. 2015). Despite these important advances in underlying mechanisms and modulating signals of plasma exosomes in modulating myocardial metabolism (Lassen et al. 2021), resisting ischemia and reperfusion injury (Vicencio et al. 2015), the effect and mechanism of plasma exosomes in myocardial ischemic angiogenesis have not been fully elucidated.

Since exosomes are vesicles generated by sequential invagination of the plasma membrane (Wei et al. 2014), tetraspanins (CD9, CD63, CD81), flotillin, Alix, etc. have been well demonstrated for the identification of exosomes (Kalluri and LeBleu 2020). CD44, which was reported to be an important regulator in membrane organization (Kumar et al. 2022; Wei et al. 2014), may play an essential role in the biogenesis and functional regulation of exosomes (Chen et al. 2022; Kelemen et al. 2022; Shen et al. 2021). CD44 was found to be widely expressed in stem cells, monocytes, tumor cells, and vascular endothelial cells (ECs), and it participates in the pathological process under ischemic conditions (Chen et al. 2020). Although the cardioprotective benefit of CD44 has been identified in alleviating adverse remodeling of the infarcted heart (Huebener et al. 2008), the regulatory role of CD44 in angiogenesis post-MI remains to be emphasized (Chen et al. 2020; Wei et al. 2014). CD44 binds and activates the growth factors, such as fibroblast growth factor 2 (FGF2) and platelet-derived growth factor-B, secreted by infiltrated monocytes to induce the establishment of collateral circulation (van Royen et al. 2004). Furthermore, the change in CD44 expression along with pathological angiogenesis and ischemia amelioration in infarct myocardium indicates the spatiotemporal correlation between CD44 and ischemic angiogenesis post-MI (Wei et al. 2014). Of note, the distribution and endocytosis of CD44, associated with CD9-positive tetraspanin-enriched microdomains and lipid rafts in microvascular ECs, present potential functional connections between CD44 and the membrane reorganization in the modulation of cell motility and angiogenesis (Wei et al. 2014). Although the pleiotropic effects exerted by CD44 in exosome biogenesis and function in cancer and chronic kidney disease have been investigated (Chen et al. 2022; Kelemen et al. 2022; Shen et al. 2021), the role of CD44 in exosomes under MI injury is still poorly understood. Given that exosomes triggering cell-to-cell communication turned out to be a major mechanism underlying the pathophysiology of MI, we proposed that the cell adhesion molecule CD44 may function as a regulator of the biogenesis of exosomes as well as the functional adjustment of exosomes in myocardial ischemic angiogenesis.

In this study, we found that CD44 expression was increased in the infarcted heart and associated with ischemic angiogenesis as well as the secretion of plasma exosomes. CD44 could affect the uptake of plasma exosomes into microvascular ECs through caveolin 1-dependent endocytosis and further regulates the plasma exosome proangiogenic ability through enhancing fibroblast growth factor receptor 2 (FGFR2) signaling transduction. Our findings demonstrate that CD44 plays a key role in plasma exosomal miRNA-enhanced angiogenic FGFR2 signaling transduction and ischemic angiogenesis in the early stage of MI.

## Methods

Detailed methods are provided in the Supplementary material.

### Animals and treatments

The CD44 knockout (KO) mice (CD44-null mice, Cat. No. 005085) purchased from Jackson Lab were mated with wildtype (WT) mice to obtain CD44 heterozygous progeny. The littermates of the heterozygous progeny were bred for multiple generations to obtain stable littermates of CD44 WT and KO mice. Eight-to-twelve week-old WT and KO mice were used for the MI model, Matrigel plug assay, and isolation of primary ECs.

For MI studies, the WT and KO mice were randomly divided into the sham groups and MI groups. The MI model was established in accordance with the guidance. In brief, mice were anesthetized under 2% isoflurane inhalation and placed on a pad in a supine position. The MI model was established by permanent ligation of the left anterior descending artery (LAD) using an 8−0 polyester suture 1 mm from the base of the left atrial appendage. Successful ligation was marked with the white anterior wall of the left ventricle and weakened cardiac function on echocardiography (left ventricular ejection fraction, LVEF < 50%). The sham groups served as the surgical controls and were subjected to the same procedures as MI mice without LAD ligation.

For the in vivo Matrigel plug assay, 8-to-12 week-old mice were injected subcutaneously with 500 µL Matrigel (Corning, 354234) supplemented with 300 ng/mL FGF2 (PeproTech, 100-18B-10). After 2 weeks, the Matrigel plugs were harvested and embedded in optimal cutting temperature (OCT, SAKURA, 4583) compound and sliced at 8 μm with a freezing microtome. After being fixed with ice acetone for 15 min, the frozen sections were blocked with 10% goat serum and incubated with CD31 rabbit antibody (CST, 77699, 1:200) overnight. The next day, the sections were incubated with Alexa 647-conjugated goat anti-rabbit antibody (Invitrogen, A32733, 1:500), followed by DAPI staining.

### Echocardiography

Cardiac function of mice was assessed with the ultrasound system GE, VIVID i with the 13 MHz transducer on day 1 and day 7 of MI. Mice were anesthetized using 2% isoflurane inhalation anesthesia and placed on a pad in the supine position. Two-dimensional M-mode tracing in parasternal short-axis view was used to assess LVEF, left ventricular fractional shortening (LVFS), left ventricular end-diastolic volume (LVEDV), and left ventricular end-systolic volume (LVESV).

### Evans blue and triphenyltetrazolium chloride (TTC) double-staining

The Evans Blue and TTC double-staining method was used to determine myocardial infarct size. Total 0.3 mL 0.5% Evans Blue (Maokangbio, MS4007) was injected into the aortic arch to identify the area at risk (AAR) 1 week after MI. The heart was rapidly excised and rinsed in normal saline solution, and then frozen at − 20 °C for 15 min. The heart was then cut into 1-mm-thick slices, which were incubated in 2% 2,3,5-TTC (Solarbio, T8170-1) at 37 °C for 30 min. The infarcted zone (IZ) (white) and the AAR (red and white) for each segment were measured with ImageJ. And the AAR/LV, IZ/AAR, and IZ/LV ratios were calculated.

### Masson’s trichrome staining

At 1 week post-MI, mice were sacrificed by an overdose of isoflurane, and the hearts were collected and fixed in 4% phosphate-buffered paraformaldehyde solution for 48 h at 4 °C. After dehydration, the specimens were embedded in paraffin. The paraffin blocks were cut into 4 μm thick microsections with Masson’s trichrome staining (Solarbio, G1340) for histological assessments. For histological quantification, multiple random microscopic fields were assessed at five different levels of the heart (ligation to apex).

### Tissue immunofluorescence

For immunofluorescence, the mouse heart tissue paraffin sections were retrieved using pH 9.0 Tris-EDTA. Then, 3% hydrogen peroxide was used to block endogenous peroxidase. The sections were incubated with primary antibodies after blocking sections using CD44 rat antibody (BD, 558739, 1:200), CD31 rabbit antibody (CST, 77699, 1:200), and cTnT mouse antibody (Huabio, EM1701-39, 1:100). Finally, sections were incubated with the secondary antibodies Alexa 647-conjugated goat anti-rabbit antibody (Invitrogen, A32733, 1:500), Alexa 647-conjugated goat anti-rat antibody (Invitrogen, A21247, 1:500), Alexa 488-conjugated goat anti-mouse antibody (Invitrogen, A32723, 1:500), and DAPI.

### Plasma and cardiac exosomes isolation

Plasma exosomes were purified through differential ultracentrifugation. In brief, blood from mice was drawn into blood collection tubes containing buffered sodium citrate as an anticoagulant. After centrifugation at 2000×*g* for 30 min at 4 °C, the plasma was systematically collected without disturbing the lower layers and then transferred to new tubes. The plasma was centrifuged at 10,000×*g* for 45 min at 4 °C to remove cell debris and microvesicles. The pellets were resuspended in 2 mL of PBS and diluted to a large volume. The suspension was filtered through a 0.22 μm filter and collected in fresh ultracentrifuge tubes at 120,000×*g* for 70 min at 4 °C. The pellets were dissolved in 50–100 µL PBS for downstream analysis. Moreover, DiD staining solution (Beyotime, C1039) was used to label exosomes according to protocol. Briefly, the isolated exosomes were stained by 5 µM DiD solution at 37 °C for 30 min. The supernatant was centrifuged at 120,000×*g* for 70 min, and then the pellets were resuspended in PBS for further tests.

The method of cardiac exosome isolation is according to a literature report (Ge et al. 2021). Briefly, mouse LV of the heart (including the border and infarcted zones) was cut into small pieces after perfusion by ice-cold PBS, and lysed by 4 mL of 0.1% type II collagenase for 30 min at 37 °C in a shaker. The lysed tissue was centrifuged at 300×*g* for 5 min at 4 °C, and then the supernatant was centrifuged at 2000×*g* for 10 min, 10,000×*g* for 10 min, and 120,000×*g* for 2 h at 4 °C (Optima MAX-XP, Beckman). The exosomes were washed with PBS and then obtained after re-centrifugation.

### Cell culture

The human umbilical vein endothelial cells (HUVECs) (CRL-1730) were purchased from ATCC, the American Type Culture Collection, and cultured in Ham’s F12k (Gibco, 21127022) and 10% fetal bovine serum (FBS, Gibco, 26010074) with 1% penicillin-streptomycin (HyClone, SV30010). HUVECs were cultured in a cell incubator at 37 °C, 5% CO_2_.

For primary mouse microvascular endothelial cells (MVECs) isolation, 8-to-12 week-old WT and CD44 KO mice were used. MVECs were isolated as previously described (Zhang et al. 2011). Briefly, cells were collected from type II collagenase-digested mouse heart, lung, and liver tissue, and enriched by CD31 mouse antibody (BD, 558736)-coated Dynabeads (Invitrogen, 11035). The cells were cultured in DMEM and 10% FBS with penicillin-streptomycin, endothelial cell supplement (Sigma, B211-GS), and heparin. Primary MVECs were used between passages two and four for assays.

For MVECs and plasma exosomes coculture studies, the plasma exosomes were quantified by using a bicinchoninic acid (BCA) kit. The working concentration of plasma exosomes was 10 µg/mL. After coculturing for 48 h, the cells were collected for further tests.

### Oligonucleotide transfection and lentivirus infection

To silence Homo CD44 expression, a custom-designed siRNA (sense strand: CGUGGAGAAAAAUGGUCGC) and negative control siRNA oligos were produced by GenePharma (China). HUVECs were transfected with CD44 siRNA (siCD44) and negative control siRNA (siControl) according to a standard protocol via Lipofectamine^®^ RNAiMAX Reagent (Invitrogen, American) for 48 h.

CD44-null HUVECs were generated with CRISPR/Cas9 technology. Single-guide RNA (sgRNA) targeting exon 2 of the CD44 locus was designed by chopchop (https://chopchop.rc.fas.harvard.edu). The sgRNA (sense strand: CTACAGCATCTCTCGGACGG) targeting CD44 was cloned into the lentiCRISPRv2 plasmid to generate the CD44-knockout construct. For lentivirus-mediated inhibition of CD44, HUVECs were grown to 60% confluency and exposed to lentiviral particles. The virus-containing medium was then removed and replaced with fresh culture medium for an additional 48 h. The cells were selected with 2 µg/mL puromycin for 2 weeks and single-cell clones were expanded.

### Immunocytochemistry

HUVECs were plated on fibronectin (10 µg/mL, Thermo Fisher, PHE0023) -coated coverglass in a 48-well cell culture plate. Cells were washed with PBS, fixed with 3% paraformaldehyde, and permeabilized with 0.1% Brij 98. Next, the cells were blocked using 10% goat serum and incubated with CD44 rabbit antibody (Proteintech, 15675-1-AP, 1:200) and FGFR2 mouse antibody (Huabio, M1501-2, 1:50) at 4 °C overnight, followed by Alexa 488-conjugated goat anti-mouse antibody (Invitrogen, A32723, 1:500) and Alexa 647-conjugated goat anti-rabbit antibody (Invitrogen, A32733, 1:500) incubation for 1 h. The cell nuclei were stained with DAPI.

Primary MVECs were cocultured with 10 µg/mL plasma exosomes within 0 min, 15 min, and 30 min. Then, the cells were fixed, permeabilized, and blocked as described above. Next, the cells were incubated with EEA1 mouse antibody (CST, 48,453, 1:100) and CAV1 rabbit antibody (Abcam, ab32577, 1:250), followed by Alexa 647-conjugated goat anti-mouse antibody (Invitrogen, A32723, 1:500) and TRITC-conjugated goat anti-rabbit antibody (Jackson ImmunoResearch, 111-025-003, 1:100) incubation. Colocalization was analyzed by Nikon NIS-Element AR Analysis software. Pearson’s correlation was used to quantify the degree of colocalization between fluorophores.

### Co-immunoprecipitation (Co-IP)

HUVECs were lysed within IP lysis buffer (Beyotime, P0013) harboring protease inhibitors for 30 min with gentle rotation at 4 °C. Supernatant lysates were collected by centrifuging at 12,000 rpm for 15 min. Equivalent protein lysates were premixed with 2 µg normal IgG rabbit antibody (Proteintech, B900610), CD44 rabbit antibody (Proteintech, 15675-1-AP), or FGFR2 rabbit (CST, 23,328) with gentle rotation at 4 °C for 1 h and then placed into incubation with nProtein G Sepharose 4 Fast Flow suspension (GE Healthcare, 17-6002-35) at 4 °C for 1 h. Resultant pellets were twice-washed with IP lysis buffer and three times with PBS, resuspended in an equal volume of 2 × loading buffer, and heated to 95 °C for 3 min on heat blocks, subsequently subjected to Western blot analysis.

### Proximity ligation assay (PLA)

PLA was used to examine CD44-FGFR2 protein interactions in situ using the Duolink^®^ In Situ Detection Reagents (Sigma, DUO92014-100RXN). HUVECs were plated on fibronectin-coated glass slides in a 48-well plate. In brief, then, slides were fixed and permeabilized, and incubated with blocking solution for 30 min at 37 °C, primary antibody CD44 Rabbit antibody (Proteintech, 15675-1-AP, 1:200), FGFR2 Mouse antibody (Abcam, ab58201, 1:50), LaminA/C Mouse antibody (CST, 4777, 1:100) and CD9 Mouse antibody (Proteintech, 60232-1-Ig, 1:200) overnight at 4 °C, PLA probe solutions for 60 min at 37 °C, Ligation-Ligase solution for 30 min at 37 °C, and Amplification-Polymerase solution for 100 min at 37 °C, sequentially. All incubations were performed without coverslips in a preheated humidity chamber. Images were obtained using confocal microscope (Nikon, N-STORM & A1) with a FITC filter for the detection of PLA signals, and analyzed by ImageJ software.

### Super-resolution imaging

Stochastic optical reconstruction microscopy (STORM) was used to examine FGFR2 distribution according to a literature report (Fu et al. 2021). Briefly, the siCD44 and siControl HUVECs were cultured in an 8-well chamber for 24 h, treated with 25 nM human FGF2 (R&D, 3718-FB) for 5 min, and washed once with PBS. After fixing and blocking the cells, the Alexa 647-conjugated FGFR2 mouse antibody (Novus, OT5C5, 1:25) was used for incubation. Cells were imaged under Nikon, N-STORM & A1 and analyzed using NIS-Elements AR software. Ripley’s K-function was used to analyze the distribution of immunolabeled proteins, the positive value of H(r) identifies clustering, and the maximum of H(r) provides a measure of domain radius (Kiskowski et al. 2009; Williamson et al. 2011).

### Western blot

HUVECs, mouse MVECs, and mouse plasma exosomes were lysed with RIPA buffer containing protease and phosphatase inhibitors, and the protein content was quantified by using a BCA kit. Protein samples were blotted according to a standard protocol. CD44 rabbit antibody (Proteintech, 15675-1-AP, 1:2000), FGFR2 rabbit antibody (CST, 23,328, 1:500), Phospho-FGFR2 (Ser782) rabbit antibody (Invitrogen, PA5-106140, 1:500), PI3k p85 rabbit antibody (CST, 4257, 1:1000), Phospho-PI3k (Tyr458/Tyr199) rabbit antibody (CST, 4228, 1:1000), Akt rabbit antibody (CST, 4691, 1:1500), Phospho-Akt (Ser473) rabbit antibody (CST, 4060, 1:1500), Erk1/2 rabbit antibody (CST, 4695, 1:1000), Phospho-Erk1/2 (Thr202/Tyr204) rabbit antibody (CST, 4370), GAPDH rabbit antibody (Sigma, G9545, 1:10000), CAV1 rabbit antibody (Abcam, ab32577, 1:2000), Alix mouse antibody (CST, 2171, 1:1000), CD63 rabbit antibody (Abcam, ab217345, 1:1000), CD81 mouse antibody (Proteintech, 66866-1-Ig, 1:1000). Finally, ImageJ software was used to analyze the immunoreactive bands.

### Cardiac tissue RNA extraction and real-time quantitative polymerase chain reaction (RT-qPCR)

The total RNA of cardiac tissue was extracted by TRIzol (Takara, 9109) method. The reverse transcription of miRNA was carried out by the stem-loop method. Reverse transcription of mRNA was performed according to the instructions provided by the reverse transcription kit (Takara, RR047A), of which the random primers were replaced with RT stem-loop primers of miRNA. The qPCR reaction was performed in a three-step method using SYBR GREEN reagent (Thermo, A25742) in the QuantStudio3 PCR system. Gene expression levels in all samples were normalized using the 2^−ΔΔCt^ method with U6 as internal controls for comparison. All primers in this study were listed in Additional file 2: Table S1.

### Statistical analysis

All data are presented as the mean ± standard error of the mean (SEM). Statistical analysis was performed using Student’s t test or one-way ANOVA. Dunnett’s post-hoc test was used for multiple comparisons between individual groups.

## Results

### CD44 promotes angiogenesis in the early stage of MI

**Fig. 1:**
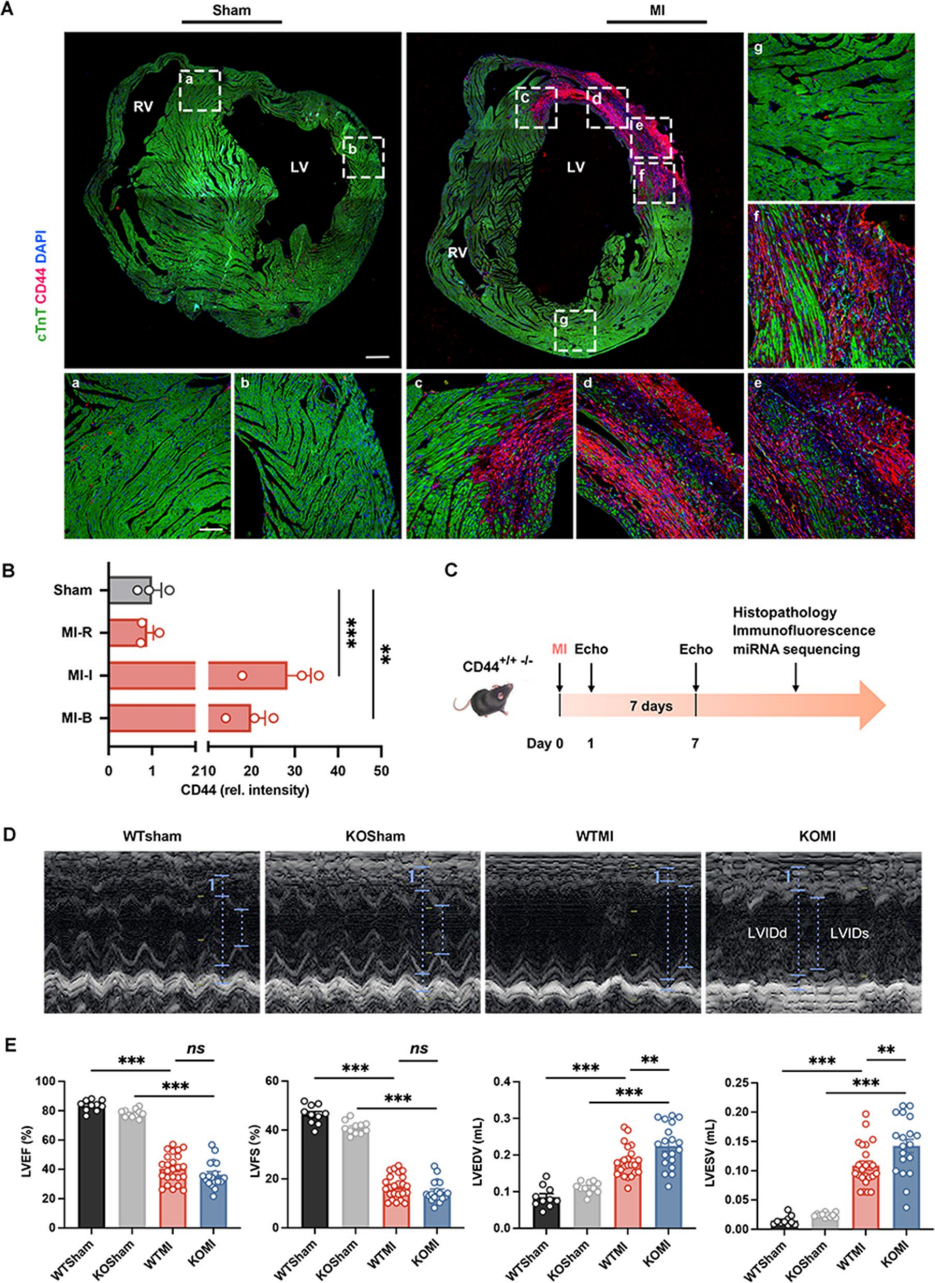
CD44 regulates cardiac remodeling and cardiac function in the early stage of MI. **A** CD44 is highly expressed in the heart in the early stage of MI. Representative confocal microscopy images of CD44 (red) expressed in cardiac border zone (c, f) and infarct zone (d, e) in 1-week MI mouse hearts and normal hearts (a, b). DAPI (blue) stained nuclei and cTnT (green) labeled cardiomyocytes. Scale bars: 400 μm (upper panel); 100 μm (lower panel). **B** The statistical data of CD44 relative fluorescence intensity in normal hearts (Sham), infarct zone (MI-I), border zone (MI-B), and remote area (MI-B) in MI mouse hearts. n = 3. **C** Schematic illustration of the mouse MI experiment. **D** The echocardiography results of the WTSham, KOSham, WTMI, and KOMI groups 1 week post-MI. LVIDd, left ventricular internal dimension at end-diastole; LVIDs, left ventricular internal dimension at end-systole. **E** The statistical data of left ventricular ejection fraction (LVEF), left ventricular fractional shortening (LVFS), left ventricular end-diastolic volume (LVEDV), and left ventricular end-systolic volume (LVESV) of WTSham (n = 10), KOSham (n = 11), WTMI (n = 24), and KOMI (n = 19) groups. *P < 0.05, **P < 0.01, ***P < 0.001, *ns* no significance. Echo, echocardiography

Remarkably, the role of CD44 in promoting pathological angiogenesis by regulating the diverse function of ECs has been confirmed (Chen et al. 2020); however, the mechanism of CD44 in ischemic angiogenesis is worth further investigation. CD44 expression was spatiotemporally correlated with angiogenesis post-MI. It is minimally detected in the normal myocardium, while its expression in the infarct zone dramatically increases along with massive cardiac angiogenesis at the early stage of MI (Huebener et al. 2008). In our study, we obtained similar results in C57BL/6 mice subjected to LAD surgery to establish a model of MI. The level of CD44 expression in the infarcted and bore zones of mouse heart tissue at 1 week post-MI was increased (Fig. 1A, B). Immunofluorescence staining demonstrated the increasing CD44 expression in the infarcted and border zones of hearts post-MI (Fig. 1A c–f), especially in vascular ECs (marked by CD31) (Additional file 1: Fig. S1 A), while the expression of CD44 in normal heart tissue of sham MI mice (Fig. 1 A a, b) or remote area of MI mice (Fig. 1 A g) was rarely detected.

Based on this observation, we also used the inbred CD44 WT and KO MI mouse model to evaluate the function of CD44 in cardiac angiogenesis and function repair (Additional file 1: Fig. S1B, C; Fig. 1C). After acute MI was established by LAD, all mice in the MI groups had left ventricle infarcts confirmed by echocardiography. The mouse hearts and plasma were collected 1 week after echocardiography (Fig. 1C). Notably, the LVEDV and LVESV of the KOMI group were larger than the those of WTMI group (Fig. 1D, E), indicating adverse ventricular contractility and predicting a high risk of cardiac death (Di Bella et al. 2021; McManus et al. 2009). Although there was no remarkable restoration in the LVEF or LVFS in the early stage of MI, a positive effect has been realized in a later stage of MI post one month (data not shown), indicating the CD44-regulated angiogenesis in the early stage could provide blood oxygen to the infarct myocardium for cell survival and cardiac function for long-term post-MI.

**Fig. 2:**
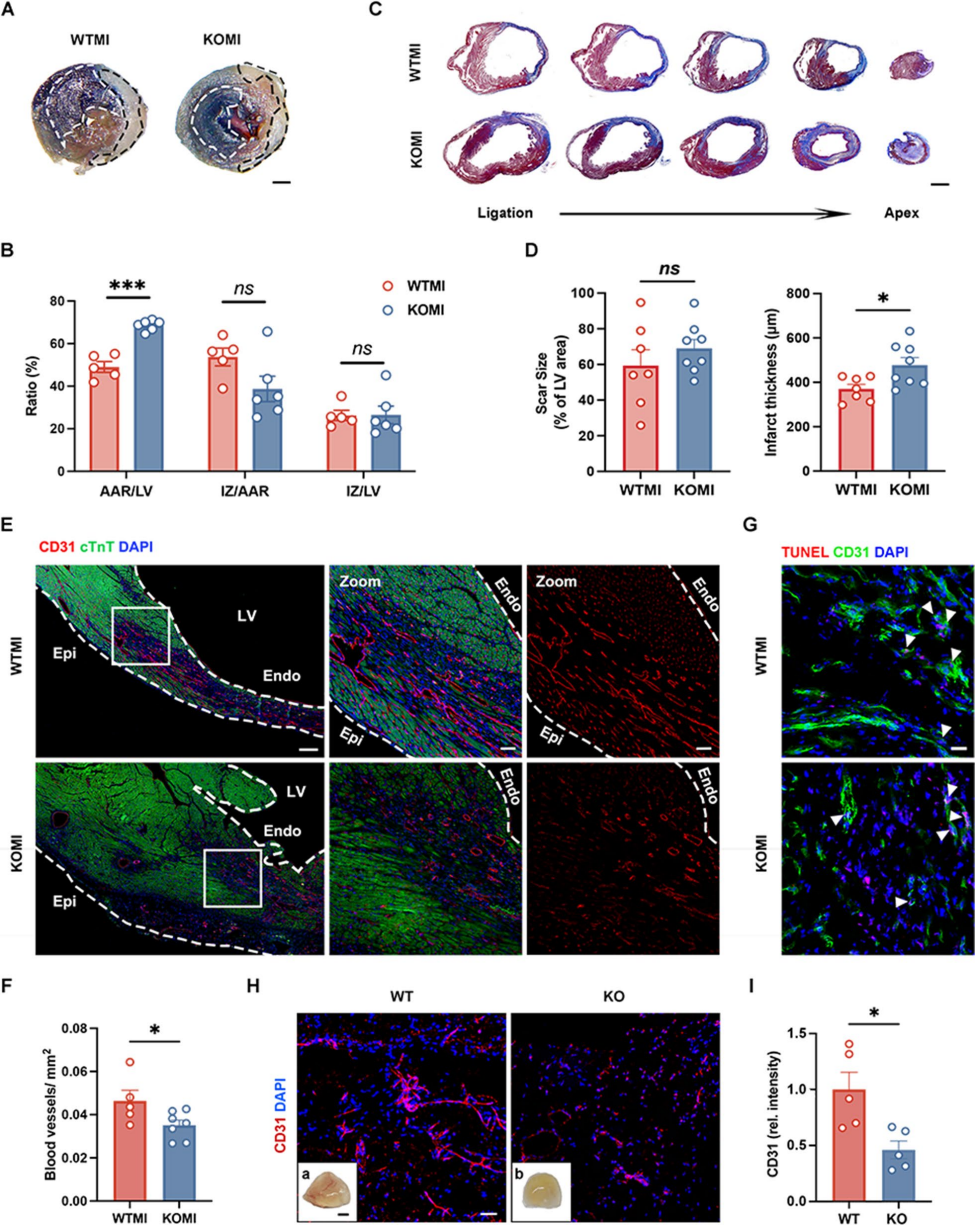
CD44 mediates cardiac ischemic angiogenesis in vivo. **A** Representative images of infarcted myocardium in CD44 WT and KO mice post 1 week MI, double-stained by Evans Blue and TTC. The black dotted line shows the area of myocardial infarction (white). The white dotted line shows the area of Evans Blue and TTC staining in LV (Blue + Red). Scale bar: 1 mm. **B** The percentages of area at risk (AAR) per left ventricle (LV), infarcted zone (IZ) per AAR, and IZ per LV in WTMI (n = 5) and KOMI mice (n = 6). **C** Representative results of Masson’s trichrome staining of heart sections from ligation to apex in the WTMI (n = 7) and KOMI (n = 8) groups. Scale bar: 1 mm. **D** Quantitative analysis of infarct size and infarct wall thickness. **E** Heart tissues were immunofluorescently stained for CD31 (endothelial marker, red), DAPI (nuclei, blue), and cTnT (green) and photographed by confocal microscopy. Scale bar: 100 μm (left panel); 10 μm (middle; right panels). **F** The statistical data of the density of blood vessels in the WTMI (n = 5) and KOMI (n = 7) groups. **G** Representative images of TUNEL (red) and CD31 (green) costaining of WTMI and KOMI mice hearts. White arrowheads denote the CD31-positive TUNEL-positive cells in cardiac border zone. Scale bar: 25 μm. **H** Immunofluorescence results of CD31 (red) staining in Matrigel plugs. Scale bar: 50 μm (right panel). DAPI (blue) labeled cell nuclei. Representative morphology of the Matrigel plugs in CD44 WT (a, n = 5) and KO (b, n = 5) mice. Scale bar: 2 mm (left panel). **I** The statistical data of CD31 relative fluorescence intensity in the WT and KO groups. *P < 0.05, ***P < 0.001, *ns* no significance. Epi, epicardium; Endo, endocardium; LV, left ventricular

Evidently, Evans blue and TTC double-staining were examined and demonstrated that the knockout of CD44 significantly improved the AAR/LV ratio 1 week after myocardium infarction compared with that of WT mice (Fig. 2A, B). Besides, the results of Masson’s trichrome staining showed that the depletion of CD44 did not influence the fibrotic scar size 1 week after MI but thickened the infarct wall, indicating that the absence of CD44 could aggravate cardiac remodeling (Fig. 2C, D). To further investigate the influence of CD44 in angiogenesis in myocardial ischemic injury, endothelial marker CD31 immunofluorescence staining was performed and revealed that CD44 KO hearts had a lower density of capillaries in the border zone than WTMI hearts 1 week after MI (Fig. 2E, F), with excluding the potential impact of endothelial cell apoptosis in CD31 density and fluorescence intensity (Fig. 2G). The finding of obvious angiogenesis in the border zone of WTMI mice consisted of the results of Evans Blue and TTC staining that verified the lower microvascular density histologically in the KO ischemic heart after infusion with Evans Blue dye (Fig. 2A, B).

To prove the role of CD44 in angiogenesis in vivo, we also performed a Matrigel plug assay in CD44 WT and KO mice by subcutaneous injection of 500 µL Matrigel mixed with 300 ng/mL mouse FGF2. After 2 weeks, we euthanized the mice and collected the Matrigel plugs. Representative photos of the WT and KO groups are shown in Fig. 2H a, b. Immunofluorescence staining showed that the relative density of blood vessels, marked by the CD31, in the CD44 KO group was significantly lower than that in the WT group (Fig. 2I), excluding the potential impact of endothelial cell apoptosis (Fig. S1 D).

As a multistep and highly regulated process, angiogenesis mainly involves the proliferation, migration, and branching of vascular ECs (Potente and Carmeliet 2017). To test the role of CD44 in angiogenesis in vitro, we compared the HUVEC viability and proliferation after siCD44 transfection via CCK-8 and EdU assays. The results showed that siCD44 led to a decrease in cell viability and proliferation (Additional file 1: Fig. S2A–C). For cell migration and invasion, Transwell cell assays were performed. There was obvious inhibition in the CD44 knockdown (KD) cells migrating or invading toward chemoattractants in comparison to the control group (Additional file 1: Fig. S2D–G). Furthermore, the branches formed by HUVECs were observed on Matrigel to determine whether CD44 mediates early morphogenetic events of ECs. The observations revealed that siCD44-treated HUVECs formed fewer tubular structures, including total segments, meshes, and nodes (Additional file 1: Fig. S2H–I).

In summary, the above results strengthened the requirement for CD44 in angiogenesis in vivo and in vitro, and CD44 acts as a crucial regulator in angiogenesis for cardiac repair in the early stage of MI.

### CD44 regulates the biogenesis and uptake of plasma exosomes post-MI

**Fig. 3:**
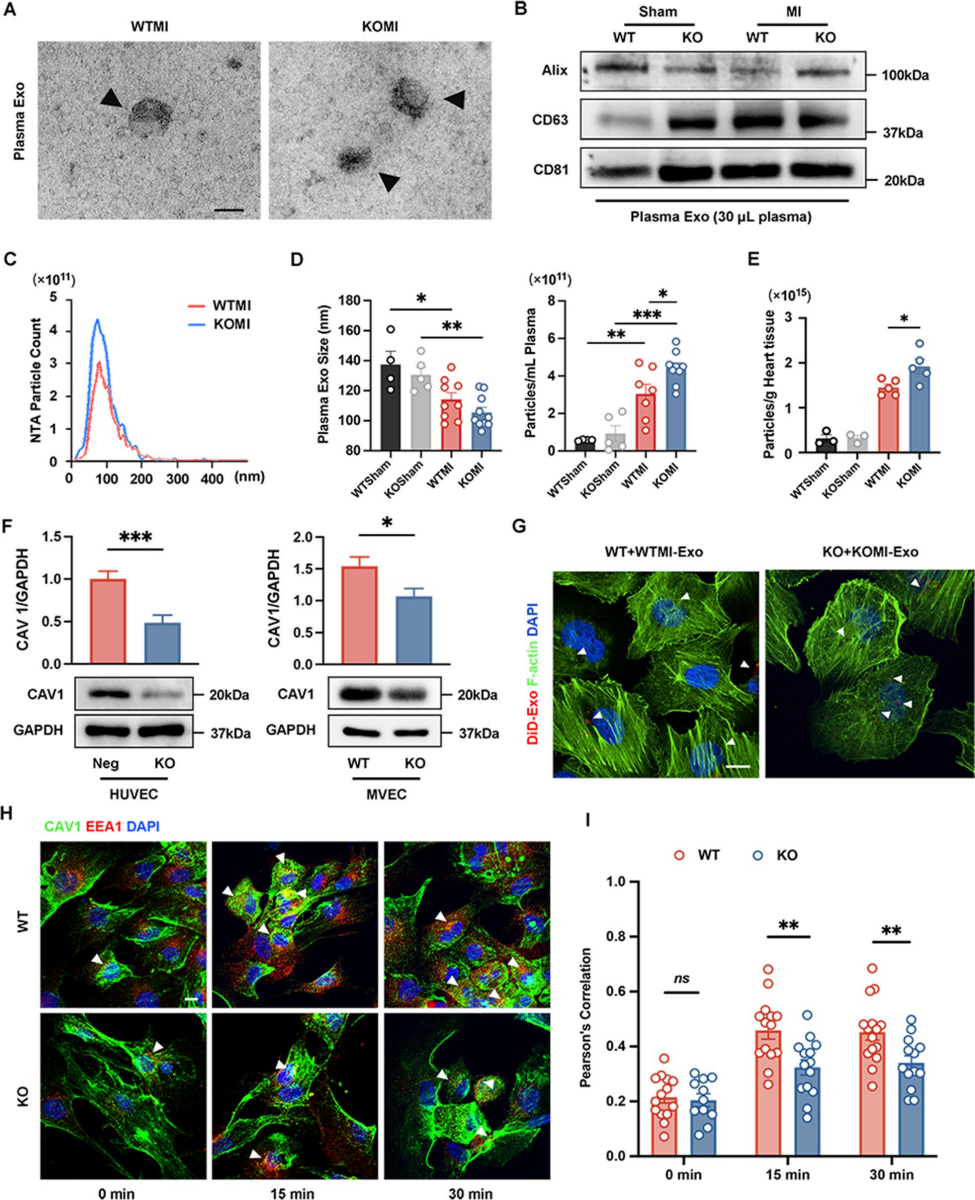
CD44 depletion impacts exosome biogenesis and weakens the CAV1-dependent exosome uptake. (A-D) The characteristics of mouse plasma exosomes 1 week after MI. **A** Transmission electron microscopy (TEM) identified WTMI and KOMI plasma exosomes. Black arrows showed exosomes. Scale bar: 100 nm. **B** Plasma exosomes extracted from the WTSham, KOSham, WTMI, and KOMI groups were examined with Western blot analysis of exosome markers (Alix, CD63, and CD81). n = 3. **C** Nanoparticle tracking analysis (NTA) identified plasma exosomes of the WTMI and KOMI groups. **D** The concentration of plasma exosomes in the WTSham (n = 4), KOSham (n = 5), WTMI (n = 9), and KOMI (n = 10) groups (left panel). The statistical data of plasma exosome sizes in the four groups (right panel). **E** Quantification of cardiac exosomes isolated from CD44 WT and KO injured LV measured using NTA. **F** Western blot analysis examined the expression of caveolin1 (CAV1) in CRISPR/Cas9-CD44 KO HUVECs and primary mouse CD44 KO MVECs. n=3. **G** Representative confocal microscopy images of the uptake of DiD-labeled WTMI-Exo and KOMI-Exo (red) by WT and KO MVECs. Phalloidin marks the cytoskeleton (F-actin, green). n = 6. DAPI (blue) stained cell nuclei. Scale bar: 10 μm. **H** Representative confocal microscopy images of plasma exosomes internalized by primary mouse MVECs within 0 min, 15 min, and 30 min. White arrowheads showed the colocalization of CAV1 (green) and the early endosome marker early endosome antigen 1 (EEA1) (red) in MVECs. DAPI (blue) stained cell nuclei. Scale bar: 10 μm. **I** The colocalization analysis of **F**. n = 11–15. *P < 0.05, **P < 0.01, ***P < 0.001, *ns* no significance

As a cell adhesion molecule, CD44 has been validated as the content transported by exosomes and mediates the functions of recipient cells in cancer (Nakamura et al. 2017; Shen et al. 2021). However, the role of CD44 in exosome biogenesis and function in MI has not yet been studied. Based on that, we collected the plasma exosomes of CD44 WT/KO sham and MI groups. Transmission electron microscopy (TEM), Western blot analysis for exosome markers, and nanoparticle tracking analysis (NTA) were utilized to identify plasma exosomes (Fig. 3A–D). The NTA results showed the mean size of exosomes from plasma was 137.5 nm in WTSham, 130.6 nm in KOSham, 114.2 nm in WTMI, and 105.5 nm in the KOMI group, showing a lessening trend of plasma exosome size post-MI in both WT and KO mice (Fig. 3D). In addition, exosome particles in plasma of two MI groups were increased compared with sham groups. This might be because the damaged myocardium can secrete exosomes into the circulatory system in response to hypoxia, and regulate the pathological process (Davidson et al. 2019). Noteworthily, the plasma exosome concentration in the KOMI group was higher than that in the WTMI group (Fig. 3D), and this finding was consistent with the tendency of cardiac particle differences in the mouse LV hearts (Fig. 3E).

As mentioned in previous studies, exosomes represent a critical effect in transferring contents and transmitting signals to elicit recipient cell functional responses after docking and uptake by cells (Barile and Vassalli 2017; French et al. 2017). Exosome uptake has been reported to occur through multiple routes that involve endocytosis by macropinocytosis and clathrin-independent pathways (Costa Verdera et al. 2017), for instance, caveolin. Because of the new insight into caveolin 1 (CAV1) in regulating endothelial barrier function and angiogenic responses (Bauer et al. 2005; Kronstein et al. 2012; Li et al. 2020), we paid more attention to CAV1. In the CRISPR/Cas9-mediated KO of CD44 HUVECs (Additional file 1: Fig. S3A) and primary mouse CD44 KO MVECs, CD44 depletion downregulated CAV1 expression (Fig. 3F). To further investigate the role of CD44 and CAV1 in endothelial endocytosis of exosomes, we cocultured mouse MVECs with mouse DiD-labeled plasma exosomes. After coculture for 12 h, two group exosomes were both internalized into WT and KO ECs (Fig. 3G). Regardless, we further detected the condition of CAV1-dependent endocytosis in two groups of MVECs by costaining CAV1 and early endosomes (marked by early endosome antigen 1 (EEA1)), where exosomes were trafficked to and released their cargo to recipient cells (Joshi et al. 2020). After coculturing for 15 and 30 min, the colocalization of CAV1 and EEA1 in KO MVECs plus KOMI-Exo was shown to be lower than that in WT MVECs plus WTMI-Exo (Fig. 3H, I). These data indicated that depletion of CD44 weakened the CAV1-dependent uptake of exosomes. Together, these findings implied that CD44 selectively controlled the CAV1-dependent uptake of exosomes in ECs.

### CD44 modulates EC function by mediating FGFR2 activation

**Fig. 4:**
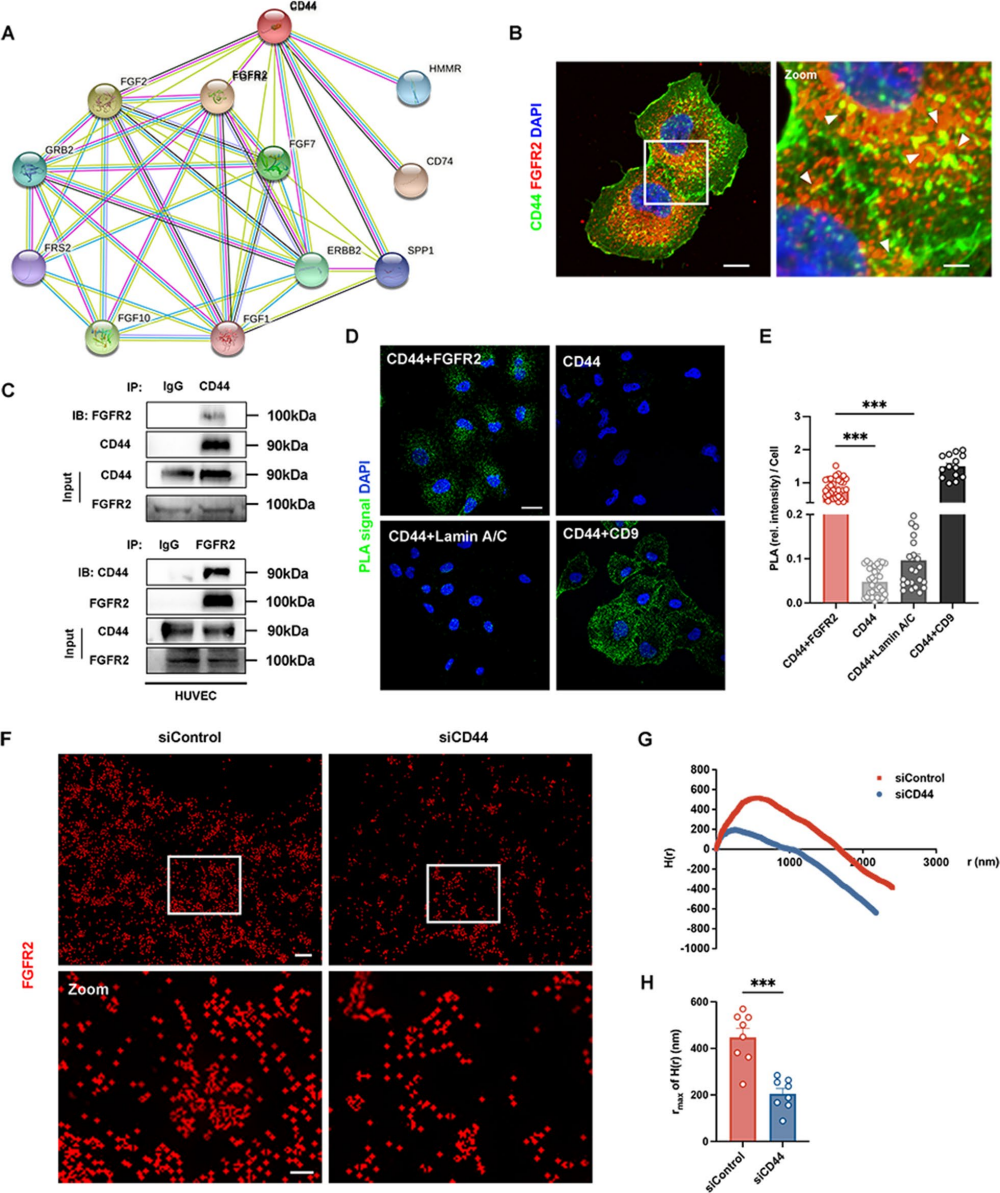
CD44 is associated with FGFR2 in ECs. **A** The STRING database was used to predict the protein-protein interaction network between CD44 and FGFR2. Pink edges represent the known protein-protein associations that are experimentally determined. Yellow edges are meant to be the predicated association via text mining. **B** Representative confocal microscopy images of CD44 (green) and FGFR2 (red) in HUVECs. Nuclei were stained with DAPI (blue). White arrowheads denote the colocalization of CD44 and FGFR2. Scale bars: 10 μm (left panel); 3 μm (right panel). **C** CD44 and FGFR2 were co-expressed in HUVECs, when the CD44 protein was immunoprecipitated by an anti-CD44 antibody, the indicated proteins, including FGFR2, were detected by immunoblotting. The same was true for the immunoprecipitation of the FGFR2 protein via an anti-FGFR2 antibody. Normal rabbit IgG acted as a negative control. **D** Representative confocal microscopy images of proximity ligation assay (PLA) signals (green) in HUVECs. CD44 served as the blank control (n = 32), CD44 + Lamin A/C served as the negative control (n = 22), and CD44 + CD9 served as the positive control (n = 14). CD44 + FGFR2 (n = 32). Scale bar: 20 μm. **E** The statistical data of **D**. **F** The FGFR2 distribution and clustering on the HUVEC cell surface were imaged at a nanoscale by N-STORM super-resolution microscopy. Scale bars: 1 μm (upper panel); 40 nm (lower panel). **G** Ripley’s H functions were acquired by NIS-Elements AR software. **H** The statistical data of the r_max_ of H(r) between the siControl and siCD44 groups. n = 8 per group. ***P < 0.001

Angiogenesis relies on extensive signal transduction between vascular ECs and other types of cells, such as cardiomyocytes, vascular smooth muscle cells, pericytes, and immune cells. Studies have confirmed that when stimulated by hypoxia, cells secrete and release proangiogenic biomolecules targeting vascular ECs, thereby activating downstream molecules to regulate cell functions (Fan et al. 2019; Katoh 2016). Among them, FGF-FGFR signaling plays an important role in angiogenesis by regulating cell migration and proliferation and affecting the morphology of blood vessels (Katoh 2016). Interestingly, the mined data from the STRING database predicted the protein interactions between CD44 and FGFR2 (Fig. 4A). Based on this finding, we next investigated the effects of CD44 inhibition on the phosphorylation and activation of FGFR2 and downstream molecules, including PI3k, Akt, and Erk1/2 in ECs. As summarized in Additional file 1: Fig. S3A–C, knockdown of CD44 potently inhibited the levels of phosphorylated FGFR2, PI3k, Akt, and Erk1/2 in HUVECs, showing that CD44 inhibition could restrain the FGFR2 signaling pathway.

Furthermore, the confocal microscopy images of immunofluorescent double-labeled staining of CD44 and FGFR2 indicated the colocalization in HUVECs (Fig. 4B). Endogenous immunoprecipitation was performed to elucidate the interactions of CD44 and FGFR2 in HUVECs (Fig. 4C). In addition, we confirmed the association between CD44 and FGFR2 on the cell membrane with PLA, which permitted the detection of the CD44-FGFR2 interaction in situ (at distances < 40 nm) at endogenous protein levels, taking CD44 and CD9 as the positive control (Fig. 4D, E). In contrast, no or a lower signal was detected in blank control (CD44 alone) and negative control (CD44 and Lamin A/C) groups (Fig. 4D, E). To determine how CD44 restrains FGFR2 activation, we measured and analyzed the FGFR2 distribution by super-resolution imaging in CD44 KD HUVECs. As expected, clustering of FGFR2 at the cell surface was markedly suppressed in CD44 KD HUVECs after treatment with FGF2 compared with control HUVECs (Fig. 4F–H). The peaks of Ripley’s H function underlined smaller sizes in FGFR2 clusters in the CD44 KD cells (Fig. 4G, H). Taken together, these findings support a positive correlation between CD44 and FGFR2 proangiogenic signaling activation.

### Plasma exosomes enhance FGFR2 signaling transduction and angiogenesis based on the regulation of CD44

**Fig. 5:**
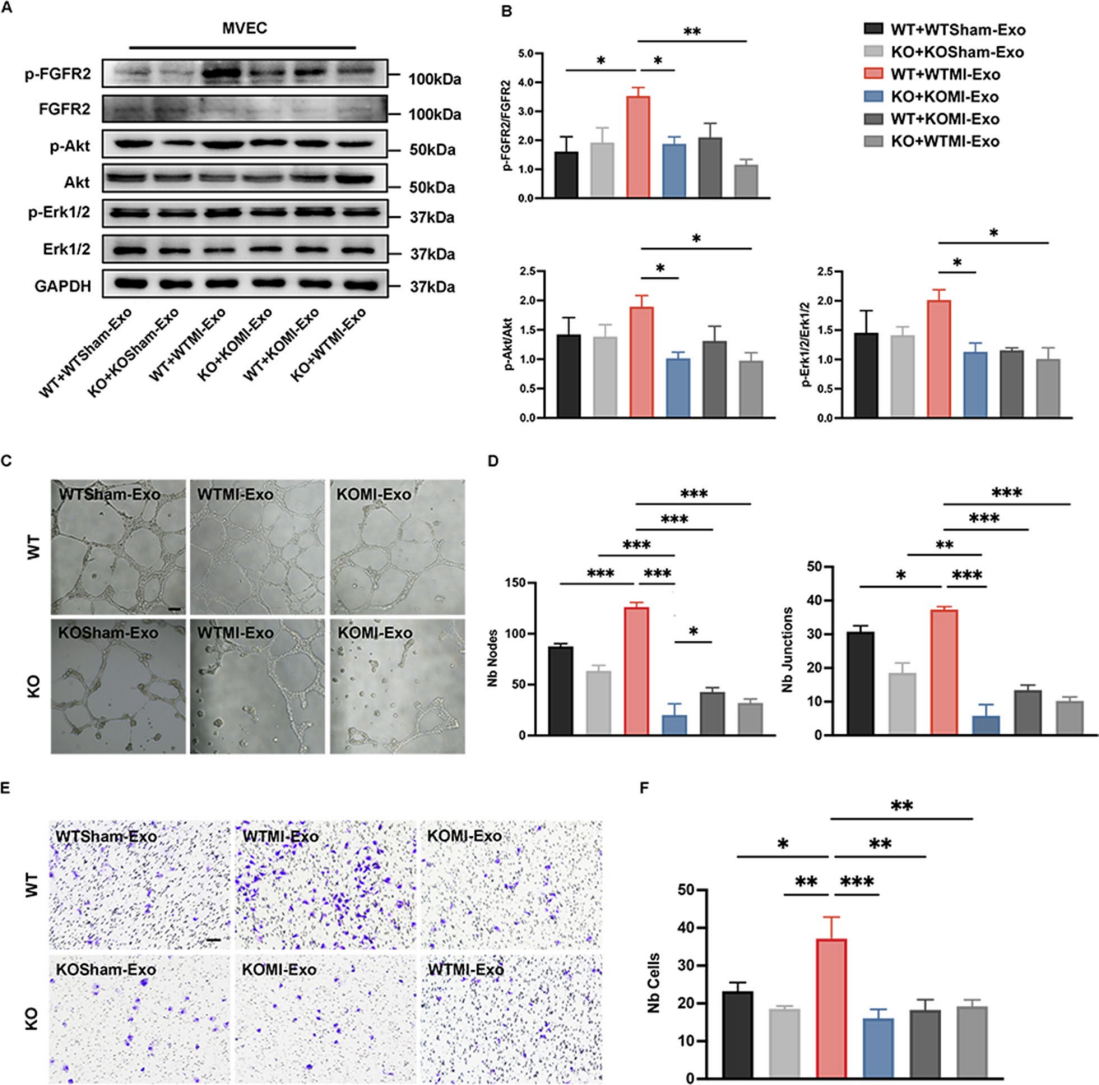
CD44 promotes plasma exosome proangiogenic function by enhancing FGFR2 signaling transduction. **A**, **B** Western blot analysis of the activation of the FGFR2 pathway after 48 h coculture of WT MVECs with WTSham plasma exosomes (WT + WTSham-Exo), KO MVECs with KOSham plasma exosomes (KO + KOSham-Exo), WT MVECs with WTMI plasma exosome (WT + WTMI-Exo), KO MVECs with KOMI plasma exosomes (KO + KOMI-Exo), WT MVECs with KOMI plasma exosomes (WT + KOMI-Exo), and KO MVECs with WTMI plasma exosomes (KO + WTMI-Exo), respectively. n = 3. **C** The tube formation assay was used to test the angiogenic function of plasma exosomes (WTSham-Exo, KOMISham-Exo, WTMI-Exo, and KOMI-Exo) in WT and KO MVECs. Scar bar: 50 μm. **D** The statistical data of the number of nodes (Nb Nodes) and the number of junctions (Nb Junctions). n = 3. **E** CD44 WT and KO MVECs were co-cultured with WTSham-Exo, KOSham-Exo, WTMI-Exo, and KOMI-Exo for 48 h, and the ability of cell migration was detected through the Transwell migration assay. Scale bar: 100 μm. **F** The numbers of cells (Nb Cells) that migrated through the inserts were counted and compared between groups statistically. n = 3. *P < 0.05, **P < 0.01, ***P < 0.001

To further establish a direct correlation between CD44-regulated plasma exosomes and myocardial ischemic angiogenesis, we tested the activation of FGFR2 signaling pathways in mouse MVECs after coculturing for 48 h. According to the results of Western blot (Fig. 5A, B), the role of CD44 in plasma exosome-triggered FGFR2 signaling was confirmed. The data showed that the phosphorylation of FGFR2, Akt, and Erk1/2 was significantly upregulated when WT ECs were cocultured with WTMI-Exo (WT + WTMI-Exo), compared with WTSham-Exo, while there was no obvious trend in KOSham-Exo and KOMI-Exo cultured with KO ECs. And the significant difference in the phosphorylation of FGFR2, Akt, and Erk1/2 between WT + WT-Exo and KO + KOMI-Exo suggested that the CD44 KOMI plasma exosomes had a weaker ability to enhance FGFR2 signaling transduction than WTMI plasma exosomes. Furthermore, when WT MVECs were treated with KOMI-Exo rather than WTMI-Exo, the enhanced effect on FGFR2 signaling activation disappeared (Fig. 5A, B). Additionally, in vitro tube formation and Transwell migration assays verified the vital role of CD44 in regulating plasma exosome-mediated proangiogenic signaling transduction. In tube formation assay, WT ECs were interconnected after coculturing with WTMI-Exo, and the tubular network was more extensive than WT ECs plus WTSham-Exo and KO ECs plus KOSham-Exo, while this overwhelming superiority could be weakened when the plasma exosomes were replaced by KOMI-Exo (Fig. 5C, D). A similar tendency was found in the Transwell migration assay (Fig. 5E, F).

**Fig. 6:**
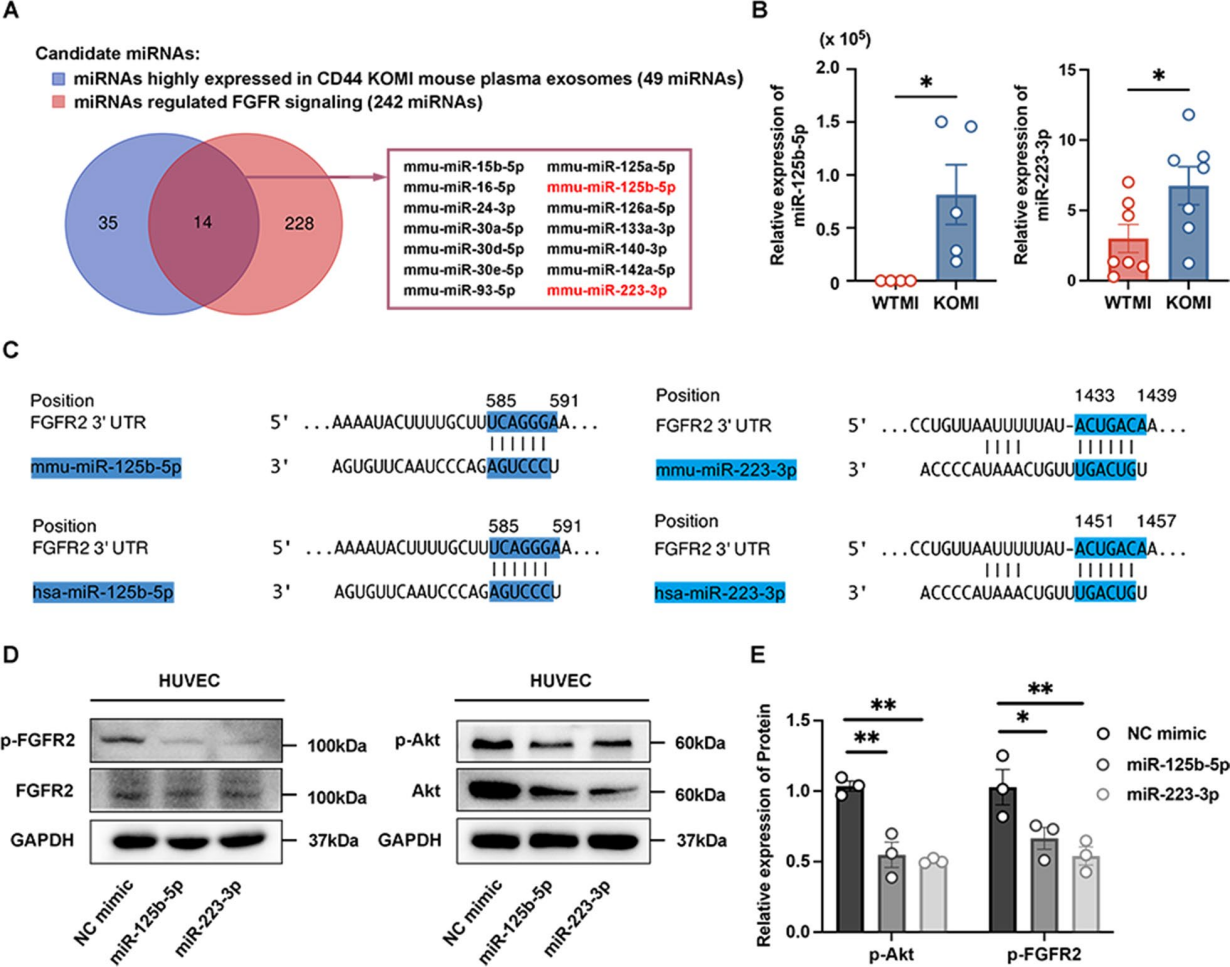
CD44 removal weakens angiogenic function of plasma exosomes by regulating miRNA-FGFR2 signaling. **A** Venn diagram of miRNAs regulating FGFR signaling pathway putatively overlapping with highly expressed in KOMI mouse plasma exosomes. **B** Real-time quantitative polymerase chain reaction (RT-qPCR) analysis of the two candidate miRNAs, miR-125b-5p and miR-223-3p, in border zone of CD44 WT and KO infarcted hearts. Data were normalized to U6 (n = 4–7). **C** TargetScan database predicated interaction between miR-125b-5p, miR-223-3p and FGFR2 3’-untranslated region (3’-UTR). **D**, **E** Western blot analysis of target genes of miR-125b-5p and miR-223-3p in the FGFR2 pathway (n = 3). *P < 0.05, **P < 0.01

The discovery of the molecular mechanism of exosomal microRNA (miRNA) facilitating angiogenesis is of utmost importance. Next, we extracted exosomes from mouse plasma for miRNA expression profiling. There were 49 miRNAs highly expressed miRNAs in CD44 KOMI mouse plasma exosomes (TPM ≥ 2000), and among that 14 miRNAs overlapping with miRNAs predicted targeting FGFR signaling pathway (GO:0008543) in mirPath v.3 and TargetScan databases (242 miRNAs) (Fig. 6A). We selected miR-125b-5p and miR-223-3p for further investigation, of which expression showed significant increasements in the CD44 KO cardiac border zone, compared with WT (Fig. 6B). It is worth noting that the miRNA database TargetScan predicted interaction between miR-125b-5p, miR-223-3p and FGFR2 3’-untranslated region (3’-UTR), which conserved among mammals (Fig. 6C). The candidate targets involved in the FGFR2 pathway were further validated in the miRNA-transfected HUVECs via Western blot analysis at the protein level. The results showed that two enforced miRNAs miR-125b-5p and miR-223-3p were able to inhibit the phosphorylation of FGFR2 and downstream molecule Akt (Fig. 6D, E). Taken together, the bioinformatics and biological experiments provide clues to plasma exosomal miRNAs regulating a series of molecules in the FGFR2 signaling pathway and angiogenesis phenotype.

**Fig. 7:**
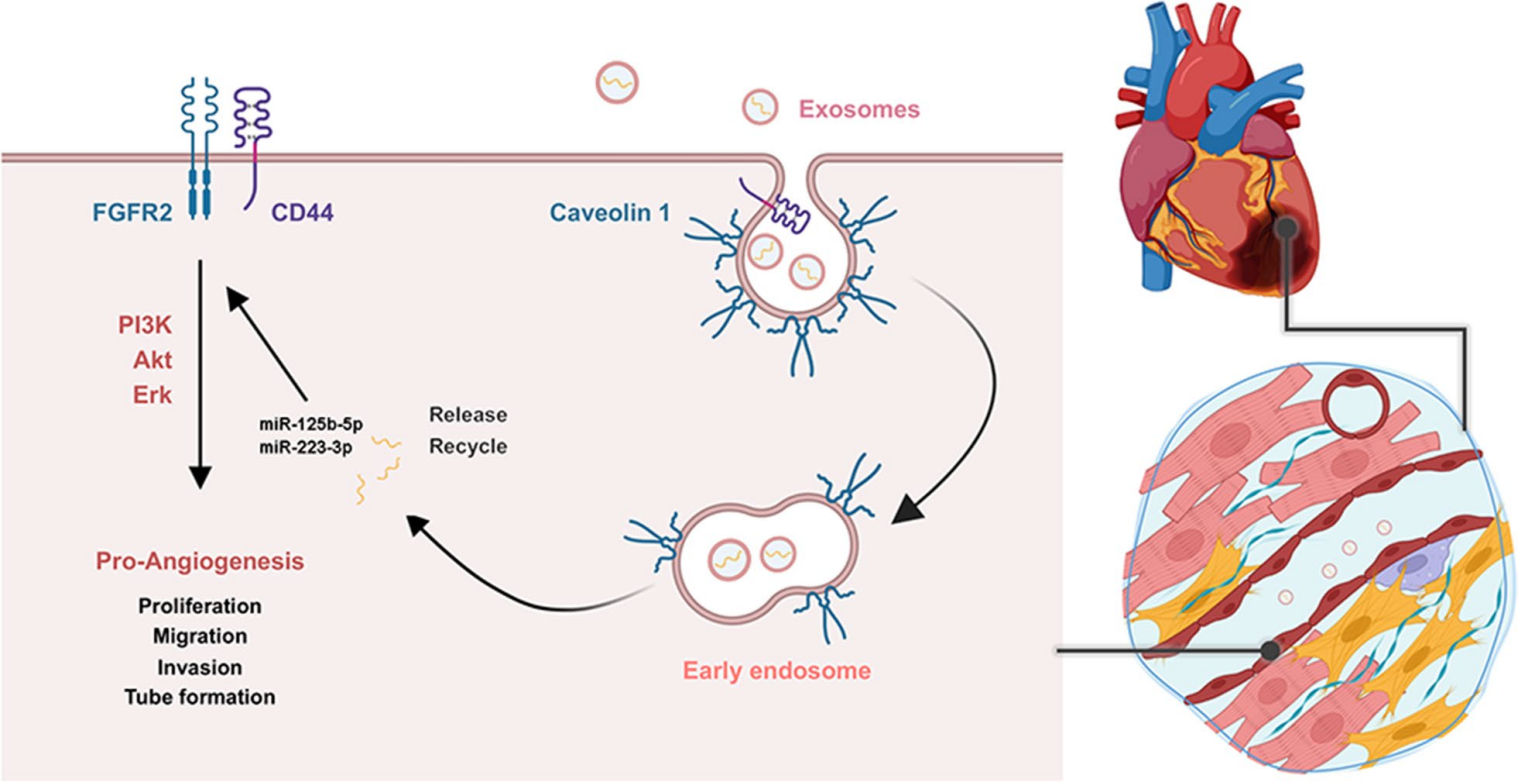
Schematic diagram of the proangiogenic mechanisms of CD44 in MI. CD44 mediates the activation of the proangiogenic FGFR2 signaling pathway in endothelial cells and regulates the CAV1-dependent uptake of plasma exosomes, of which the contents miR-125b-5p and miR-223-3p enhance the FGFR2 signaling pathway to promote angiogenesis post-MI

Collectively, these findings demonstrated that CD44 is of great significance for the proangiogenic effect of plasma exosomes through enhancing FGFR2 signaling transduction. CD44 mediates the activation of the proangiogenic FGFR2 signaling pathway in endothelial cells post-MI, and regulates the CAV1-dependent uptake of plasma exosomes, the contents, miR-125b-5p and miR-223-3p, which enhance the FGFR2 signaling pathway to promote angiogenesis. A schematic diagram of the proangiogenic mechanisms of CD44 in MI is shown in Fig. 7.

## Discussion

The development and repair of the heart are inseparable from angiogenesis. After the occurrence of AMI, the vascular endothelial cells that exist in the infarcted area receive external signals, which lead to proliferation, migration, sprouting, tube formation, and maturation of vessels (Wu et al. 2021). The neonatal blood vessels bring nutrients to the infarcted zone and rescue the damaged and dying heart tissue (Wu et al. 2021). Since reconstruction and restoration of blood supply have been considered to be the main strategies in the treatment of coronary heart disease, the “therapeutic angiogenesis” concept has been proposed as a new revascularization strategy in recent decades (Losordo and Dimmeler 2004a, 2004b; Mitsos et al. 2012). Emerging evidence has mentioned that the cell adhesion molecule CD44, which was well demonstrated to regulate cell proliferation, adhesion, and migration, plays an important role in angiogenesis (Chen et al. 2020; Jaskuła et al. 2021; Savani et al. 2001), both in the tumor angiogenesis (Cao et al. 2006; Ludwig et al. 2019) and ischemia angiogenesis (Chen et al. 2020). Previous studies have pointed out that CD44 can regulate the processes of inflammation, fibrosis, and cardiac remodeling after myocardial ischemia (Huebener et al. 2008), but few studies have demonstrated the relationship between CD44 and angiogenesis following MI. Nevertheless, the therapeutic potential of CD44 in pathological angiogenesis is emerging (Chen et al. 2020) and in our previous study, we also reported that CD44 was presented as a potential therapeutic target to intervene in angiogenesis under pathological conditions (Wei et al. 2014). Hence, we proposed the crucial role of CD44 in pathological angiogenesis post-MI, and in this study, we mainly aimed to identify CD44 as a crucial regulator involved in ischemic angiogenesis and explore the mechanism. The in-depth insights into pathological angiogenesis in myocardial ischemia may inspire better therapeutic strategies.

Based on our current results, we identified that CD44 was expressed at low levels in normal mouse hearts, which was consistent with the findings in the Human Protein Atlas database (https://www.proteinatlas.org): relative low expression of CD44 on cardiomyocytes and vascular endothelial cells in normal human heart tissue. Nevertheless, CD44 was upregulated in the myocardial infarcted and border zones, especially in CD31-positive MVECs 1 week after the onset of MI in mice. Therefore, we realized that CD44 may not be necessary for angiogenesis under physiological conditions but is crucial for pathological angiogenesis following MI and may serve as a biomarker of early angiogenesis after AMI, leading to a positive effect on impaired cardiac function in a later stage of MI. The existence or overexpression of CD44 may potentiate the effect of novel therapeutic angiogenesis in MI treatment.

As an important cell membrane protein, CD44 was reported to functionally regulate the organization of EC membrane proteins and associate with the exosome marker CD9 (Wei et al. 2014). Accordingly, it is not surprising that CD44 may participate in regulating the biogenesis of exosomes and even modulating their function. A large number of studies have confirmed the feasibility and effectiveness of therapeutic angiogenesis strategies for cardiovascular disease, and exosome therapy is well underlined (Boulanger et al. 2017; Sahoo and Losordo 2014). Exosomes are known to be used as carriers to encapsulate protein, DNA, RNA, miRNA, etc., and deliver biosignals into recipient cells that regulate the status of cell signaling pathways and the cell phenotypes (Kalluri and LeBleu 2020). Indeed, the protective characteristics of circulating exosomes have attracted significant interest in MI treatment, and enthusiastic attempts about the application of plasma exosomes have been made in cardioprotection (Davidson et al. 2019; Femminò et al. 2020). A previous study has shown that as a membrane protein, CD44 and variant CD44v6 were engaged in exosome biogenesis, loading, and delivery in cancer-initiating cells (Wang et al. 2018). In addition, it was reported that CD44-enriched exosomes can mediate tumor cell motility and promote cancer metastasis by enhancing the uptake efficacy of exosomes by target cells (Nakamura et al. 2017; Shen et al. 2021). As noted, except for the crucial effects of CD44 in exosome biogenesis and therapeutic function in cancer, the role of CD44 in exosome biogenesis and its angiogenic function in MI injury have not been well elucidated and deeply discussed.

From this perspective, we detected a higher concentration of plasma exosomes in CD44 KOMI mice, as well as a similar tendency of cardiac exosomes in the LV of the heart. Although there is no evidence to clarify the direct effect of the number of circulating exosomes in the development of MI, there is certainly a large number of circulating exosomes with heterogeneities of size, content, source, and function after MI (Barile et al. 2017; Davidson et al. 2019; Kalluri and LeBleu 2020). It should be emphasized that, according to our study, CD44 exerts effects on the number and size of plasma exosomes. As suggested by analyses of the energetics and thermodynamics, membrane spontaneous curvature and stiffness play key roles in the formation of physiological extracellular vesicles and vesicle size distribution, such as exosomes (Huang et al. 2017). Moreover, a recent discovery uncovered that the transmembrane protein CD9, highly associated with CD44, also works as a curvature sensor with a preference for positive membrane curvature (Dharan et al. 2022). Herein, it is reasonable to assume that CD44 may serve as a collaborator in protein-driven shaping and remodeling of membranes. Consequently, lacking CD44 leads to a compensatory increase and size variation in circulating exosomes post-MI.

It is well accepted that circulating exosomes encounter and bound to the recipient cell surface, then be internalized into cells partially via endocytosis commonly (O’Brien et al. 2020). The endocytic process such as the uptake of exosomes in ECs is partly regulated by caveolae-mediated endocytosis (Costa Verdera et al. 2017; Filippini and D’Alessio 2020; French et al. 2017; Yang et al. 2021), among that abundant CAV1 is a critical component (Filippini and D’Alessio 2020). In our study, a relatively lower expression of CAV1 has been found with CD44 knockout in HUVECs and primary mouse MVECs, indicating the potential association between CD44 and CAV1 in ECs. Although a previous study has elicited a noticeable regulatory effect on CAV1-dependent membrane organization and *C. neoformans* internalization in human brain MVECs (Long et al. 2012), to date, there is no evidence supporting the correlation between CD44 and CAV1 in exosome uptake. Most prominently, we tested the stronger CAV1-dependent exosome uptake and enriched localization in early endosomes for cargo recycling in the presence of CD44. Accordingly, we demonstrated that the uptake of plasma exosomes, which involves CAV1-dependent endocytosis, may occur in a CD44-mediated manner in ECs. Meanwhile, it has been observed that the uptake fluorescence intensity of a single cell showed no significant difference in WT and KO MVECs after coculturing with WTMI-Exo and KOMI-Exo for 12 h, respectively. It has been summarized the transfer of exosomal cargoes to the recipient cells could occur through several mechanisms (Corbeil et al. 2020). Although CD44 knockout affects CAV1-mediated endocytosis, other forms of endocytosis could compensate for the impact. Nevertheless, this conclusion requires more direct and convincing evidence in future investigations.

As described above, clarifying the underlying mechanism of CD44 in the regulation of therapeutic angiogenesis will contribute to MI treatment. Due to the central role of growth factors in therapeutic angiogenesis, the association between CD44 and growth factor-induced angiogenic signals has undoubtedly attracted much attention. In addition to the obvious effects of CD44 in regulating the angiogenic phenotype of ECs, some studies demonstrate the positive relationship between the expression of CD44 and proangiogenic growth factors, such as VEGF, FGF2, and so on (Liao et al. 2022; Ludwig et al. 2019). Indeed, there have been observations indicating CD44 coordinates with VEGF to promote tumor and ischemia-induced angiogenesis. CD44 and CD44v6, presenting a critical role of VEGF-VEGFR signaling transduction, were involved in the proliferation and angiogenesis of ECs (Ludwig et al. 2019; Tremmel et al. 2009); after treating with VEGF, the formation of blood vessels and CD44 expression were enhanced in rats post-MI (Garcia et al. 2015). Although observations have unearthed the essential role of VEGF, angiogenesis therapies focused on VEGF-VEGFR alone have limitations and other angiogenic factors such as FGF are also crucial (Vimalraj 2022). Remarkably, FGFR2, as a keystone growth factor receptor, takes charge of the induction and enhancement of angiogenesis (Fan et al. 2019; Katoh 2016). Although it has been proposed that FGFR2 is coexpressed and colocalized with CD44 in gastric cancer cells (Park et al. 2016), it is still unclear whether the association between these two proteins exists in ECs. Herein, the data presented here are focused on the correlation between CD44 and FGF-FGFR signaling pathway. More intriguingly, an important finding in this study is that CD44 directly associates with FGFR2 on HUVECs and affects the transduction of FGFR2 signaling, which given that the inhibition of CD44 could desensitize ECs to FGF2 induction as well as the FGFR2 signaling transduction.

On the other hand, for decades, it has been noted that one specific function of circulating exosomes is closely related to the different amounts of their contents, such as miRNA exosomal cargo (Barile et al. 2017). When the profile and amount of miRNA exosomal cargo varied, the biosignals that were delivered to recipient cells changed accordingly (Kalluri and LeBleu 2020), resulting in different functional outcomes in response to hypoxia stimulation (Barile et al. 2017; Cheng et al. 2019). In our experiment, we observed that plasma exosomes could carry diverse cargo molecules, including miRNAs, to recipient cells MVECs, where they afforded a remarkable protective effect against MI injury in the presence of CD44, especially in promoting and enhancing the angiogenic function of ECs, while the proangiogenic effects of plasma exosomes on ECs were weakened by depletion of CD44. In this context, direct administration of proangiogenic plasma exosomes of the mouse groups with/without CD44 to MVECs presented the diverse activation states of the FGFR2 signaling pathway intriguingly.

Notably, elucidating the underlying effects of CD44 in regulating plasma exosome-induced angiogenesis indeed contributes to clarifying the mechanism of exosome-based therapeutic angiogenesis post-MI. The miRNA profiling and bioinformatics analysis of plasma exosomal contents allowed the discovery of a brand-new role for miR-125b-5p and miR-223-3p in regulating the FGFR2 pathway and angiogenesis. As mentioned above, activation of the FGFR2 pathway is one of the most important events that initiate angiogenesis in ECs in response to the stimulation of angiogenic FGFs (Fan et al. 2019; Katoh 2016). Our results showed that key molecules involved in the FGFR2 pathway, including FGFR2, Akt, and Erk1/2, are promoted by the CD44 WTMI-Exo in ECs, while miR125b-5p and miR-223-3p enriched in KOMI-Exo suppressed the initiation and transduction of the FGFR2 pathway. This finding further provided solid evidence demonstrating that CD44-regulated plasma exosomes were indispensable for FGFR2 signaling transduction.

### Perspectives

Given the evidence in exosome-based therapeutic angiogenesis against MI injury and based on our research findings, in this study, we raised the prospects aiming to deeply investigate therapeutic angiogenesis in MI, including exosome-based therapy. Over the past decades, the therapeutic potential of exosome therapy in pathological angiogenesis in myocardial ischemia has been confirmed. Although therapeutic exosome therapy has good application prospects, it also encounters obstacles, such as the challenge of manipulating the biodistribution of endogenous exosomes (Liu et al. 2020) and the unsatisfactory efficacy of vascular delivery and uptake (Hu et al. 2021). Therefore, enhancing the cardiac targeting ability and facilitating biological effects of cardioprotective exosomes for clinical therapy as needed. To date, several studies have aimed to overcome these bottleneck problems (Hu et al. 2021; Liu et al. 2020; Segura-Ibarra et al. 2017). For instance, the application of hyaluronic acid (HA), a ligand of CD44, in nanomedicine has shown a promising perspective in targeted therapy (Lee et al. 2020; Lei et al. 2021). Since studies on cancer and renal ischemia mentioned the function of HA-CD44 in nanoparticle-based strategies for satisfactory efficacy (Awadalla et al. 2021; Chen et al. 2021), HA-loaded exosomes or nanoparticles may bring out the promise for the application in MI treatment. Combined with the findings of the increasing change in CD44 expression that we observed in the mouse cardiac infarcted and border zones, we hold the opinion that HA-loaded nanoparticles or exosomes may target the damaged heart tissue efficiently by binding to the highly expressed HA receptor CD44. Furthermore, cell cytokines, including the growth factor FGF2, could be encapsulated in nanoparticles or engineered exosomes to fully exerted the proangiogenic effect through FGFR2 signaling with the collaboration of CD44.

## Conclusion

The present study reveals an intriguing phenomenon in which CD44 regulated-plasma exosomes exert angiogenic effects in ECs by enhancing the activation of the FGFR2 signaling pathway not only in the presence of CD44 but via its content of miRNAs. It can be concluded that CD44 is essential in promoting FGFR2-induced angiogenesis, in particular enhancing plasma exosomal miRNA-mediated FGFR2 signaling transduction in myocardial ischemic angiogenesis.

## Supplementary information

**Additional file 1**: **Supplementary methods and figures of this study. Fig. S1** CD44 is highly expressed in the heart in the early stage of MI, especially in microvascular endothelial cells.  **Fig. S2** CD44 knockdown inhibits the angiogenic function of HUVECs. **Fig. S3** The proangiogenic FGFR2 signaling pathway is suppressed in the depletion of CD44.

**Additional file 2: Table S1. **PCR primers used in this study.

## Data Availability

The datasets generated during and/or analyzed during the current study are available from the corresponding author on reasonable request.

## Abbreviations

MI: Myocardial infarction
ECs: Endothelial cells
FGF2: Fibroblast growth factor 2
FGFR2: Fibroblast growth factor receptor 2
KO: Knockout
WT: Wildtype
LAD: Left anterior descending artery
LVEF: Left ventricular ejection fractions
LVFS: Left ventricular fractional shortening
LVEDV: Left ventricular end-diastolic volume
LVESV: Left ventricular end-systolic volume
HUVECs: Human umbilical vein endothelial cells
MVECs: Microvascular endothelial cells
TEM: Transmission electron microscopy
NTA: Nanoparticle tracking analysis
PLA: Proximity ligation assay
SEM: Standard error of the mean
KD: Knockdown
CAV1: Caveolin 1
EEA1: Early endosome antigen 1
miRNA: microRNA
