# Supplementary material for: CD44 promotes angiogenesis in myocardial infarction through regulating plasma exosome uptake and further enhancing FGFR2 signaling transduction
Source: Mol Med. 2022 Dec 3;28:145. doi: 10.1186/s10020-022-00575-5 (PMC9719212; doi:10.1186/s10020-022-00575-5)
Supplement: Supplementary file 1 — Additional file 1: Supplementary methods and figures of this study. Fig. S1 CD44 is highly expressed in the heart in the early stage of MI, especially in microvascular endothelial cells. Fig. S2 CD44 knockdown inhibits the angiogenic function of HUVECs. Fig. S3 The proangiogenic FGFR2 signaling pathway is suppressed in the depletion of CD44. [file 10020_2022_575_MOESM1_ESM.docx]

**Methods**

**Mouse Genotyping**

The tails of 3-4-week-old mice were lysed using protease K and tail lysate at 55 °C heat. Centrifuge at high speed for 10 min to settle impurities, add NaCl and isopropanol sequentially to the supernatant, precipitate DNA, and wash the DNA multiple times with 70% ethanol. After high-speed centrifugation, add 50 μL of ddH_2_O to resuspend the extracted DNA. Finally, touch-down polymerase chain reaction (PCR) and agarose gel electrophoresis were performed. All primers in this study were listed in Supplemental Table 1. No template control (NTC) is set to confirm whether primer dimers are produced and whether there is template contamination in the system.

**TUNEL And CD31 costaining**

The apoptosis of endothelial cells in MI hearts and Matrigel plug were detected by TUNEL and CD31 costaining. TUNEL (Elabscience, E-CK-A322) staining was performed after the frozen sections were fixed with acetone and treated with proteinase K. Next, frozen sections were blocked with 10% goat serum, and incubated with CD31 rabbit antibody (CST, 77699, 1:200) and the secondary antibody Alexa 488-conjugated goat anti-rabbit antibody (Invitrogen, A32723, 1:500), and DAPI.

**Transmission electron microscopy (TEM) imaging**

Exosomes were imaged through TEM. In brief, 20 μL of the fresh sample of exosomes that were not subjected to freezing temperatures at any step during processing was loaded on carbon-coated copper electron microscopy grids for 2 min. The samples were then negatively labeled with phosphotungstic acid solution for 5 min. The grids were washed thrice with PBS to remove extra phosphotungstic acid solution and maintained under semidry conditions using filter paper. Images were obtained with a TEM (Hitachi, H7650) at 80 kV.

**Nanoparticle tracking analysis (NTA)**

The size distribution and concentration of exosomes were analyzed using a NanoSight NS300 system equipped with a 405 nm laser. In brief, 10 μL of exosomes with 990 μL of 0.22 μm filtered sterile PBS was pushed slowly using a 1 mL syringe and illuminated with a laser. Their movements under Brownian motion were recorded in 30-sec sample videos, which were analyzed with NTA analytical software (NanoSight, version 3.0). At least three videos were captured for each sample. The capture and analysis settings were manually set in accordance with the manufacturer’s instructions.

**Exosome RNA extraction and quality control**

Total RNA was isolated and purified using TRIzol following the manufacturer's procedure. Quality was measured using Agilent 2100 Bioanalyzer (Agilent, G2939AA) and NanoPhotometer® (Implen, N60).

**miRNA library construction and sequencing**

Ten ng of total miRNA was used as input for library preparation with the NEXTflex® Small RNA-Seq Kit v3(Bio Scientific Corporation, NOVA-5132-05) following the manufacturer's instructions. Adapters are ligated sequentially to the 3' and 5' ends of miRNAs in an unbiased reaction. miRNAs with adaptors were reverse-transcribed to generate cDNA with reverse transcription reagent. After purification by NEXTflexTM Cleanup Beads (Bio Scientific Corporation, NOVA-5132-05), the ligated products were amplified with PCR by the following conditions: initial denaturation at 95℃ for 2 min; 22-25 cycles of denaturation at 95℃ for 20 sec, annealing at 60℃ for 30 sec, and extension at 72℃ for 15 sec; and then final extension at 72℃ for 2 min. The average insert size for the final cDNA library was 155 ± 5 bp.

Subsequently, the library was purified by NEXTflexTM Cleanup Beads and assessed using Agilent 2100 Bioanalyzer (Agilent, G2939AA) and NanoPhotometer ® (Implen N60). Finally, library sequencing was performed on an Illumina Novaseq™ 6000 (Illumina Corporation San Diego U SASA) platform following the vendor's recommended protocol by Guangzhou Huayin Health Medical Group CO., Ltd. (Guangzhou, China).

**miRNA annotation analysis**

Clean reads were mapped to the genome (Mus musculus) using Bowtie (http://bowtie-bio.sourceforge.net/index.shtml, v0.12.9). sRNAs were mapped to miRBase, Rfam, snoRNA, and piRNA databases using Bowtie to identify known miRNAs. Mirdeep2 (https://www.mdc-berlin.de/content/mirdeep2-documentation, v2.0.0.5) was used to predict new miRNAs that were not annotated on any database above and aligned to the antisense strands of the genomic exon, introns, and intergenic regions. A self-made program was used to calculate the expression of sRNA by counting corresponding transcripts per million (TPM).

**Data extraction**

The STRING database (http://string-db.org) was used to predict the protein-protein interaction network between CD44 and FGFR2. Human CD44 protein distribution information in different tissues including heart was obtained in The Human Protein Atlas (https://www.proteinatlas.org). The miRNA target prediction was performed with DIANA Tools mirPath v.3 (https://dianalab.e-ce.uth.gr/html/mirpathv3/index.php?r=mirpath) and TargetScan (http://www.targetscan.org/). The Venn diagram was drawn using the website (http://bioinformatics.psb.ugent.be/webtools/Venn/).

**miRNA Transfection**

When the HUVECs density was 60%, mimics NC (UUCUCCGAACGUGUCACGUTT), miR-125b-5p mimic (AGUGUUCAAUCCCAGAGUCCCU), and miR-223-3p mimic (ACCCCAUAAACUGUUUGACUGU) produced by GenePharma (China) were transfected with Lipofectamine® RNAiMAX Reagent (Invitrogen, American). Total Protein was extracted 48 h after transfection.

**Cell viability and proliferation assay**

HUVECs were plated in 96-well plates, and we used the CCK8 assay to detect cell viability. Working solution with 10 μL CCK8 (Beyotime, C0042) was added to every well after siRNA transfection and incubated for 1 h at 37°C. We measured the absorbance (OD value) using Spectrophotometer at 450 nm. For the EdU assay, reagent A was added to the 96-well plate with the medium and incubated at 37°C for 2 h to label EdU. Cells were treated and stained following the manufacturer’s instructions (RiboBio, C10310-1). Finally, the cells were imaged with a fluorescence microscope (Nikon, Ni-E).

**Migration assay**

The migration of HUVECs and MVECs was analyzed using the Transwell assay. After treatment, 8×10^3^ starving cells per well were seeded in the top Transwell chamber with an 8.0 μm pore polycarbonate membrane insert and cultured in 300 μL of F12k medium with 0.1% BSA. The bottom chambers were filled with 500 μL of medium with 1% FBS. After 16 h of migration, the cells inside the upper chamber were wiped with cotton swabs, and the migrated cells were fixed with 3% paraformaldehyde for 15 min. After washing with PBS, the cells were stained with 1% crystal violet for 30 min. At least five random visual fields were captured under microscope (Nikon, SMZ18) at 10× magnification.

**Invasion assay**

For the invasion assay, after treatment, 3×10^4^ cells per well were seeded in the top Transwell chamber (8.0 μm) gelled with Matrigel (1:30 dilution in F12k) in 300 μL of 1% FBS with 1% FBS. The bottom chambers were filled with 600 μL of F12k with 5% FBS. After 24 h of invasion, the cells inside the upper chamber were wiped with cotton swabs, and the migrated cells were fixed with 3% paraformaldehyde for 15 min. After washing with PBS, the cells were stained with 1% crystal violet for 30 min. At least five random visual fields were captured under microscope (Nikon, SMZ18) at 10× magnification.

**Tube formation assay**

Matrigel was thawed at 4 ℃, pipetted 50 μL/well into 96-well plates, and placed at 37°C for 30 min for gelling. After treatment, 3×10^4^ cells (100 μL of F12k medium) were seeded into wells. The network of tubes was photographed after 12 h under the microscope at 4× magnification, and the total segments length, node, junctions, and meshes of tubes were analyzed using ImageJ software.

**Figures**


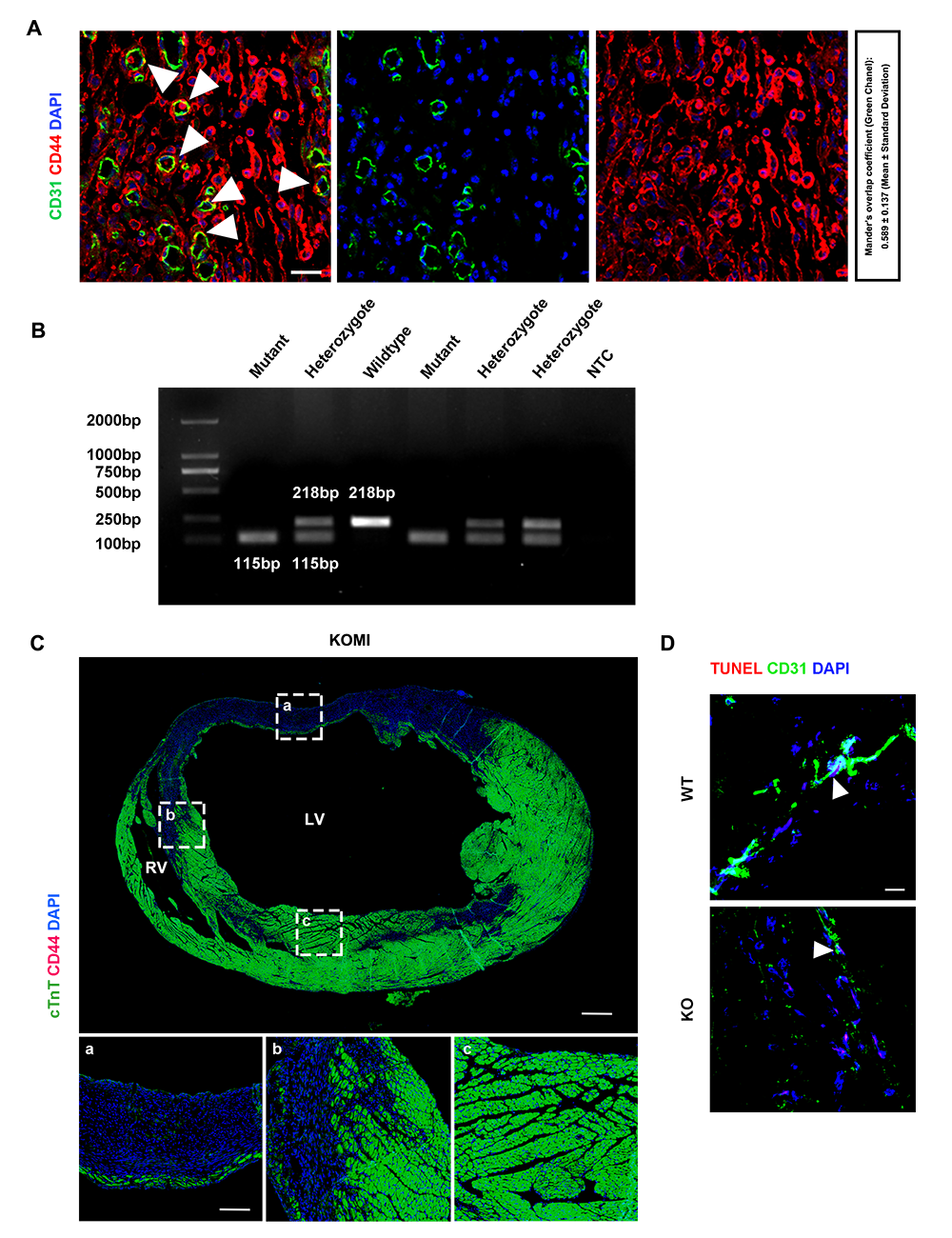


Fig.S1 CD44 is highly expressed in the heart in the early stage of MI, especially in microvascular endothelial cells. (A) CD44 (red) was located in MVECs in the heart border zone. Scale bars: 25 μm (left panel); 10 μm (right panel). White arrowheads denote the location of CD44 in MVECs. DAPI (blue) stained nuclei and CD31 (green) labeled endothelial cells. The ratio of CD31/CD44-double-positive vs CD31 was analyzed by Mander’s overlap coefficient (Green Chanel-CD31). n=5. (B) Genotyping results of CD44 wildtype (CD44 WT) = 218bp, CD44 mutant (CD44 KO) = ~115bp, and CD44 heterozygote = ~115bp and 218bp. (C) Representative confocal microscopy images of CD44 KOMI mice with no expression of CD44 (red) in heart. DAPI (blue) stained nuclei and cTnT (green) labeled cardiomyocytes. Scale bars: 400 μm (upper panel); 100 μm (lower panel). (D) Representative confocal microscopy images of TUNEL and CD31 staining of Matrigel plug sections. White arrowheads denote the location of TUNEL signals in MVECs. Scale bars: 20 μm. NTC: No template control.


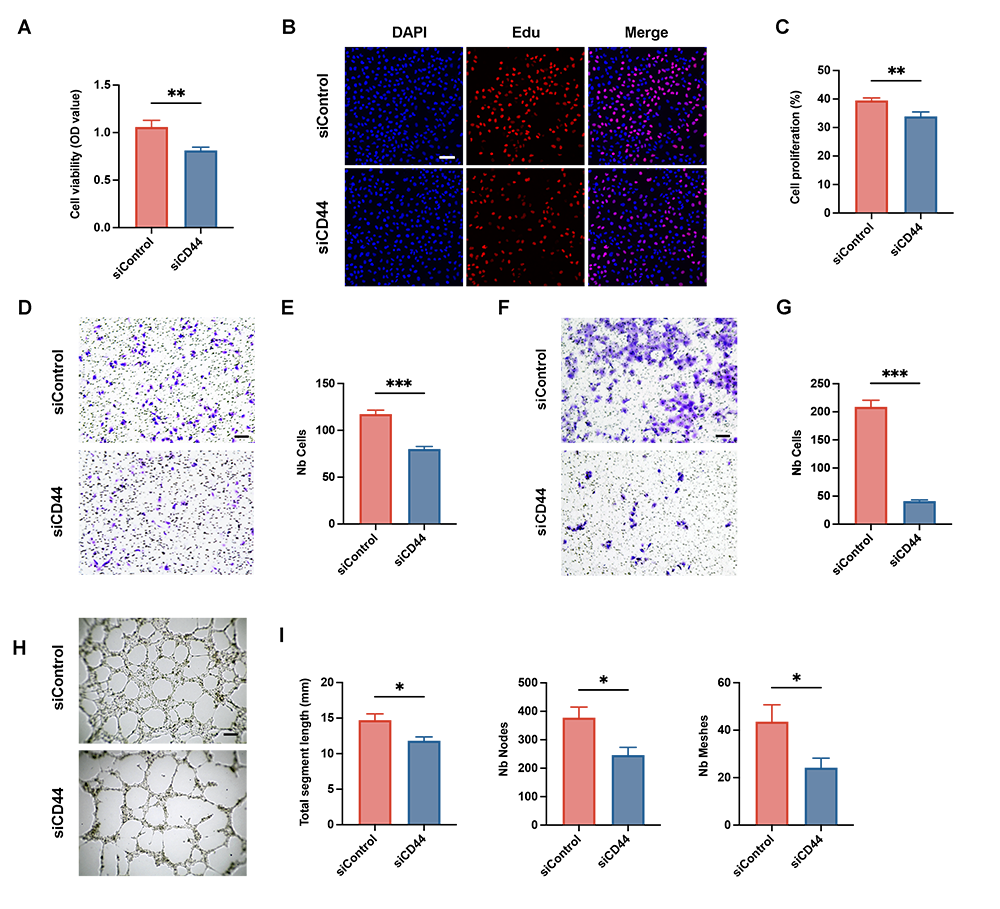


Fig.S2 CD44 knockdown inhibits the angiogenic function of HUVECs. (A-C) CD44 removal inhibited HUVEC viability (A) and proliferation (B, C). (B, C) Photomicrographs and quantification analysis of EdU-labeled (red) HUVECs. DAPI (blue) stained nuclei. Scale bar: 200 μm. (D-G) Cell migration and invasion abilities were measured via Transwell migration and invasion assays. Scale bars: 100 μm. (H, I) Tube formation assay results, including the total segments length, number of nodes (Nb Nodes), and number of meshes (Nb Meshes) determined the tube formation ability in HUVECs. Scale bar: 100 μm. n=3. * *P*<0.05, ** *P*<0.01, *** *P*<0.001.


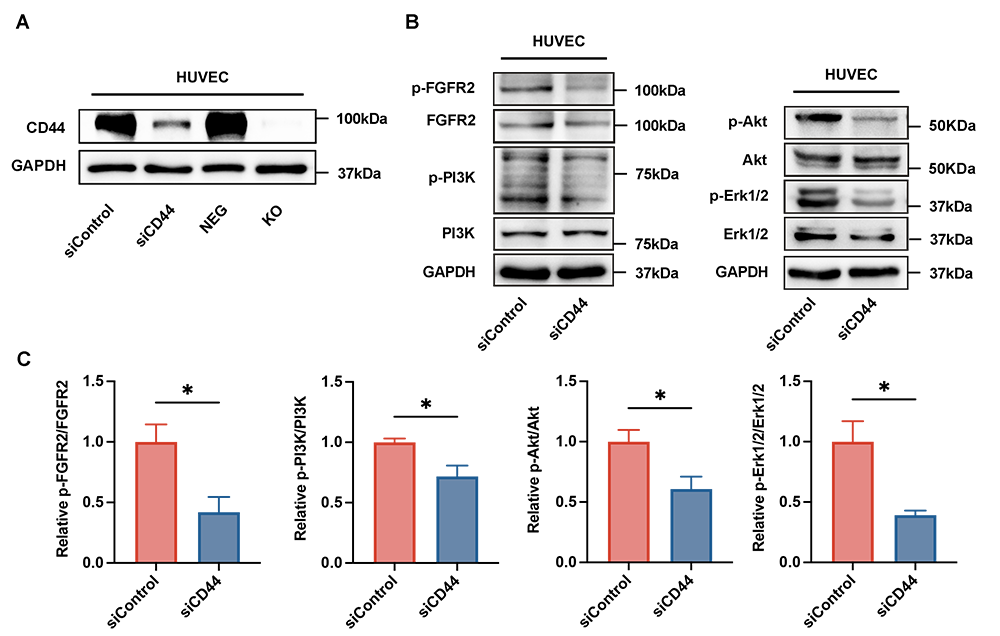


Fig.S3 The proangiogenic FGFR2 signaling pathway is suppressed in the depletion of CD44. (A) Western blot confirmed the efficacy of CD44 knockdown and knockout via siCD44 transfection and the CRISPR/Cas9 system, respectively. (B) Western blot analysis measured the activation of the FGFR2-PI3k-Akt/Erk1/2 signaling pathway in HUVECs. (C) The statistical data of (B). n=3. * *P*<0.05, ** *P*<0.01, *** *P*<0.001.
