# Supplementary material for: CD44 promotes angiogenesis in myocardial infarction through regulating plasma exosome uptake and further enhancing FGFR2 signaling transduction
Source: Mol Med. 2022 Dec 3;28:145. doi: 10.1186/s10020-022-00575-5 (PMC9719212; doi:10.1186/s10020-022-00575-5)
Supplement: Supplementary file 2 — Additional file 2: Table S1. PCR primers used in this study. [file 10020_2022_575_MOESM2_ESM.docx]

Supplementary Table 1. PCR primers used in this study.

| **Genotyping Primer 5’ - 3’** | |
| --- | --- |
| Common | GCGACTAGATCCCTCCGTTT |
| Wildtype Reverse | ATCCCAGCTTTGCTTTGCTA |
| Mutant Reverse | GTTTTCCCAGTCACGACGTT |
| **miRNA RT-qPCR Primer 5’ - 3’** | |
| Mouse U6 Forward | TGGAACGCTTCACGAATTTGCG |
| Mouse U6 Reverse | GGAACGATACAGAGAAGATTAGC |
| mmu-miR-125b-5p RT | GTCGTATCCAGTGCAGGGTCCGAGGT  ATTCGCACTGGATACGACTCACAA |
| mmu-miR-125b-5p Forward | CGCGTCCCTGAGACCCTAAC |
| mmu-miR-223-3p RT | GTCGTATCCAGTGCAGGGTCCGAGGT  ATTCGCACTGGATACGACTGGGGT |
| mmu-miR-223-3p Forward | GCGCGTGTCAGTTTGTCAAAT |
| Common miRNA Reverse | AGTGCAGGGTCCGAGGTATT |
